# Severity-stratified vasopressor-escalation strategies in septic shock: a cross-fitted doubly robust analysis with external replication

**DOI:** 10.3389/fphar.2026.1876555

**Published:** 2026-07-23

**Authors:** Yuhang Zhang, Menglin Han, Xiaosu Liu, Guanyu Chen, Lulu Zhang

**Affiliations:** 1 Department of Anesthesiology, The Second Affiliated Hospital of Chongqing Medical University, Chongqing, China; 2 Department of Critical Care Medicine, The Second Affiliated Hospital of Chongqing Medical University, Chongqing, China; 3 Department of Anesthesiology, Zigong Fourth People’s Hospital, Zigong, Sichuan, China

**Keywords:** doubly robust estimation, heterogeneous treatment effects, septic shock, target trial emulation, vasopressors

## Abstract

**Background:**

The optimal vasopressor-escalation strategy after norepinephrine in septic shock remains uncertain, with randomized trials showing no clear winner and observational data suggesting severity-dependent effects. We characterized severity-stratified heterogeneity for adding vasopressin or epinephrine to norepinephrine and tested external replicability.

**Materials and Methods:**

We emulated a target trial in Medical Information Mart for Intensive Care IV (MIMIC-IV) (derivation) and eICU Collaborative Research Database (eICU-CRD) (external validation), enrolling adults with Sepsis-3 septic shock who started norepinephrine. Patients were classified as S1 (norepinephrine alone), S2 (plus vasopressin), or S3 (plus epinephrine) by the agent added in a baseline window. The primary outcome was 14-day administratively truncated in-hospital mortality. Average treatment effects were estimated with a cross-fitted doubly robust learner, anchored on a prespecified norepinephrine-equivalent dose below 0.25 μg/kg/min, with subgroups, sensitivity analyses, and E-values. A depth-2 policy tree summarized heterogeneity, not a decision rule.

**Results:**

After trimming, 2,415 MIMIC-IV and 568 eICU-CRD patients were analyzed. Cohort-level effects were heterogeneous and did not replicate: in MIMIC-IV, S3 *versus* S1 was −9.66 percentage points (95% confidence interval −19.36 to −0.11) and S2 *versus* S1 was null (+1.60; −7.79 to +5.47), whereas in eICU-CRD the S3 contrast reversed (+0.73; −26.10 to +28.44). At the low-dose anchor, S3 *versus* S1 was −10.87 pp (−19.66 to +2.97) in MIMIC-IV and directionally consistent but imprecise in eICU-CRD (−13.72; −48.24 to +9.47); the anchor E-value of 2.19 exceeded plausible unmeasured confounding. The S2 contrast was the one most exposed to confounding by indication (E-value 1.24).

**Conclusion:**

Vasopressor-escalation effects were heterogeneous and concentrated at low baseline severity, with a directionally consistent but unconfirmed signal favoring epinephrine below the Surviving Sepsis Campaign dose threshold. These findings are hypothesis-generating and do not support changing first-line practice; future trials should stratify on baseline norepinephrine-equivalent dose rather than enroll uniform cohorts.

## Introduction

Sepsis caused an estimated 21.4 million deaths worldwide in 2021, with a sustained reversal of the decline observed before the COVID-19 pandemic (GBD 2021 Global Sepsis Collaborators, 2025). Septic shock—the subset with circulatory and metabolic dysfunction—carries in-hospital mortality exceeding 40% (Shankar-Hari et al., 2016). Vasopressor selection is among the few early-management decisions that clinicians make hourly at the bedside and among the few without a clear evidence-based answer.

Norepinephrine is the recommended first-line vasopressor (Evans et al., 2021), but the evidence for second-line escalation is fragmented. Neither major randomized trial of adjunctive vasopressin was conclusive: VANISH found no kidney-failure-free-day benefit yet could not exclude clinically important effects (Gordon et al., 2016), and a VASST secondary analysis suggested a survival benefit in less severe shock that was never confirmed (Russell et al., 2008). The 2021 Surviving Sepsis Campaign (SSC) guideline accordingly only makes a weak suggestion to add vasopressin at norepinephrine doses of 0.25–0.5 μg/kg/min—a threshold drawn from expert practice rather than randomized evidence (Evans et al., 2021) and the one we adopt below as the prespecified severity anchor. Evidence for adding epinephrine is even sparser: no adequately powered trial has compared norepinephrine plus epinephrine with norepinephrine alone using mortality as the primary endpoint.

Two recent studies have sharpened the timing question. The OVISS reinforcement-learning study derived a vasopressin-initiation rule from 3,608 patients and reported lower mortality in adherent cohorts (Kalimouttou et al., 2025), and a target trial emulation by White et al. (2025) found a comparable direction of benefit for vasopressin started within 6 h of shock, with effect modification by norepinephrine-equivalent dose. Neither study compares the three escalation strategies that clinicians actually choose among within a single framework that resolves how their effects vary with severity.

We used MIMIC-IV (Johnson et al., 2023) and eICU-CRD (Pollard et al., 2018) to compare three vasopressor-escalation strategies, namely, norepinephrine alone, norepinephrine plus vasopressin, and norepinephrine plus epinephrine, for in-hospital mortality in adults with septic shock. Time zero was norepinephrine initiation, and the strategy was assigned by the agent added within a prespecified early window thereafter (Methods). We hypothesized that the better strategy depends on shock severity at the time of escalation and that no single strategy dominates across the cohort and specified a norepinephrine-equivalent dose below 0.25 μg/kg/min—the SSC threshold noted above—as the primary severity anchor. Effects were estimated with a cross-fitted doubly robust (DR) learner (Athey and Wager, 2021), and a depth-2 policy tree fitted to the resulting scores gave a structural summary of the estimated heterogeneity, presented as hypothesis-generating rather than as a clinical decision rule. The pipeline was rerun in eICU-CRD as external replication, with sensitivity analyses including E-values.

## Materials and methods

### Study design and data sources

**FIGURE 1:**
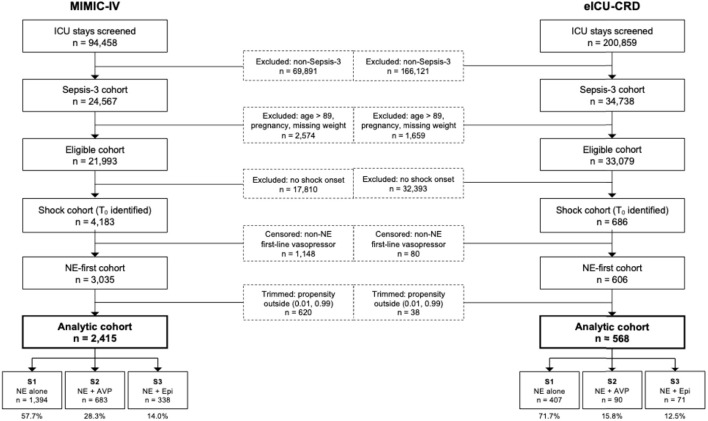
Cohort derivation in MIMIC-IV and eICU-CRD. Parallel flow diagrams of cohort derivation for both databases, with sequential exclusions shown in dashed boxes and the analytic cohort split by strategy at the bottom. In MIMIC-IV, 94,458 ICU stays were screened; 69,891 did not meet Sepsis-3 criteria; 2,574 of the remaining 24,567 patients were excluded for age above 89 years, pregnancy, or missing weight; 17,810 of the 21,993 eligible patients did not develop septic shock; 1,148 of the 4,183 shock patients (T_0_ identified) received a non-NE first-line vasopressor and were censored; and 620 of the 3,035 NE-anchored patients were trimmed for estimated propensity outside [0.01, 0.99], yielding an analytic cohort of 2,415 split into S1 (NE alone; n = 1,394, 57.7%), S2 (NE + AVP; n = 683, 28.3%), and S3 (NE + Epi; n = 338, 14.0%). In eICU-CRD, 200,859 ICU stays were screened and the same sequential exclusions (166,121; 1,659; 32,393; 80; 38) yielded an analytic cohort of 568 split into S1 (n = 407, 71.7%), S2 (n = 90, 15.8%), and S3 (n = 71, 12.5%). AVP, vasopressin; eICU-CRD, eICU Collaborative Research Database; Epi, epinephrine; ICU, intensive care unit; MIMIC-IV, Medical Information Mart for Intensive Care IV; NE, norepinephrine; S1, NE alone; S2, NE plus AVP; S3, NE plus Epi; T_0_, time zero of septic-shock onset.

We used MIMIC-IV v3.1 (Johnson et al., 2023) and eICU-CRD v2.0 (Pollard et al., 2018) in a target trial emulation following Hernán and Robins (2016). MIMIC-IV and eICU-CRD served as the derivation and external replication cohorts, respectively. Secondary analysis of these de-identified databases is exempt from institutional review at our institution. Cohort derivation is shown in Figure 1.

### Eligibility and time zero

Adults (≥18 years) with a first ICU admission and first sepsis episode were eligible. Sepsis was defined by Sepsis-3 criteria (Singer et al., 2016) using the five-component Sequential Organ Failure Assessment (SOFA) score (mSOFA-5, with the cardiovascular component omitted; see Supplementary eMethods S1). Septic shock was defined as concurrent mean arterial pressure (MAP) < 65 mmHg and lactate >2 mmol/L, conditional on either ≥30 mL/kg crystalloid administered in the preceding 6 h or initiation of any acute vasopressor for ≥1 h (Supplementary eMethods S1). Time zero (T_0_) was the first hour at which all three conditions were met.

Eligibility required initiation of norepinephrine (NE) within [T_0_, T_0_+3 h], serving simultaneously as the cohort-entry criterion and the strategy-assignment window to satisfy the no-anticipation requirement of target trial emulation (Hernán and Robins, 2016). Patients receiving an alternative first-line agent (most often phenylephrine in post-anesthesia contexts or dopamine, vasopressin, or epinephrine without NE) were excluded from the three-arm comparison; these excluded patients are described in Supplementary eTable S1. Patients with do-not-resuscitate orders, transitions to comfort care, or palliative-care planning prior to T_0_ were also excluded.

### Treatment strategies

By eligibility, all patients received NE as their first-line vasopressor. We compared three vasopressor-escalation strategies, distinguished by the second-line decision made within an early-resuscitation window of [T_0_, T_0_+3 h]:

S1 (NE-only): NE without addition of vasopressin (AVP) or epinephrine in the window
S2 (NE + AVP): NE with AVP in the window, no epinephrine
S3 (NE + epinephrine): NE with epinephrine added in the window, with or without AVP

Exposure required a non-zero infusion rate at any of the four hourly observations within the window. This intention-to-treat-style assignment captures the decision to add an adjunctive agent regardless of subsequent dose titration (Hernán and Robins, 2016). Norepinephrine-equivalent dose (NEE) was computed using established conversion factors: 1:1 for NE and epinephrine and vasopressin 0.03 U/min ≈0.1 μg/kg/min at a 70 kg reference weight (Wieruszewski and Khanna, 2022) (Supplementary eMethods S2). The distribution of NEE at T_0_ across strategies is shown in Supplementary eFigure S1.

### Confounders, outcome, and missing data

Twenty-four baseline covariates were extracted at T_0_ or summarized over the preceding 6 h: demographics (age, sex, race, weight, and body mass index [BMI]); pre-existing comorbidities (heart failure, chronic kidney disease ≥ stage 3, chronic liver disease, and diabetes); severity at T_0_ (mSOFA-5, MAP, lactate, NEE, mechanical-ventilation status, and the peripheral oxygen saturation to inspired oxygen fraction [SpO_2_/FiO_2_] ratio) and trajectories over 6 h pre-T_0_; time intervals from infection to organ dysfunction and to T_0_; and empirical antibiotic class. Detailed definitions are listed in Supplementary eMethods S3 and pairwise correlations in Supplementary eFigure S2.

The primary outcome was 14-day administratively truncated in-hospital mortality: death in hospital within 14 days of T_0_, the horizon set by the administrative follow-up window common to both databases. Discharge alive (home, rehabilitation, or skilled-nursing facility) and continued admission at day 14 were coded as alive; interhospital transfers and discharges against medical advice were handled by inverse-probability-of-censoring weighting (Supplementary eMethods S3). Discharges to hospice were coded as alive in the primary analysis and as deaths in a sensitivity analysis.

Missingness exceeded 20% only for BMI, lactate trajectory features, and—in eICU-CRD—MAP trajectory features (Supplementary eFigure S3); these reflect measurement frequency rather than the outcome, justifying a missing-at-random assumption. The primary analysis used single iterative-regression imputation, with multiple imputation by chained equations (MICE) prespecified as a sensitivity analysis (Buuren and Groothuis-Oudshoorn, 2011). Imputation procedures and the MAR justification are detailed in Supplementary eMethods S3.

### Statistical analysis

Causal contrasts were estimated by a cross-fitted doubly robust (DR) learner (Chernozhukov et al., 2018; Athey and Wager, 2021). This is a point-treatment estimator—a single escalation decision at T_0_ with a single end-of-follow-up outcome—in which the propensity and outcome nuisance models and the target parameter are estimated on independent fivefold partitions (“cross-fitting”); it is not a time-varying or longitudinal (“sequential”) estimator. On each fold, two nuisance components were fit: a multinomial logistic propensity model and a per-strategy L2-regularized logistic outcome model. L2 logistic regression was selected over gradient-boosted models for better small-subset calibration (Supplementary eFigure S4); gradient-boosted models were retained as a sensitivity specification. Model formulations and hyperparameter tuning are given in Supplementary eMethods S4.

The DR pseudo-outcome combined the outcome regression with an inverse-propensity correction term (Athey and Wager, 2021). Patients with any propensity outside [0.01, 0.99] were trimmed for positivity, ensuring adequate covariate overlap across strategies for valid counterfactual contrasts; the trimmed cohort is described in Table 1. Average treatment-effect contrasts (S2 minus S1, S3 minus S1) were computed as mean differences of DR scores across the trimmed sample, with 95% confidence intervals and two-sided p-values from 2,000 bias-corrected and accelerated (BCa) bootstrap resamples (seed 42). The same full-pipeline bootstrap, refitting both nuisance models on every resample, was identically applied in MIMIC-IV and eICU-CRD, such that interval construction is harmonized across the two cohorts. Bootstrap protocol and a cross-implementation numerical check are described in Supplementary eMethods S5. As a point-treatment emulation, the design adjusts for confounding measured at and before T_0_ but does not model post-assignment time-varying confounding (e.g., later titration or agent changes); this restriction is revisited in the Discussion.

**TABLE 1:** Baseline characteristics of the trimmed septic-shock cohort by vasopressor strategy.

Characteristic	MIMIC-IV (n = 2,415)				eICU-CRD (n = 568)			
	S1 (n = 1,394)	S2 (n = 683)	S3 (n = 338)	*p*	S1 (n = 407)	S2 (n = 90)	S3 (n = 71)	*p*
Demographics	​	​	​	​	​	​	​	​
Age, years	68.0 (57.0, 77.0)	65.0 (55.0, 75.0)	69.0 (57.0, 77.0)	<0.001	68.0 (58.5, 77.5)	65.5 (54.2, 75.0)	68.0 (57.0, 75.5)	0.151
Sex, male, n (%)	822 (59.0%)	407 (59.6%)	207 (61.2%)	0.744	214 (52.6%)	58 (64.4%)	45 (63.4%)	0.048
Race/ethnicity, n (%)	​	​	​	0.028	​	​	​	0.300
White	889 (63.8%)	400 (58.6%)	205 (60.7%)	​	293 (72.0%)	69 (76.7%)	53 (74.6%)	​
Black	132 (9.5%)	71 (10.4%)	29 (8.6%)	​	39 (9.6%)	4 (4.4%)	4 (5.6%)	​
Asian	39 (2.8%)	26 (3.8%)	4 (1.2%)	​	4 (1.0%)	0 (0.0%)	0 (0.0%)	​
Hispanic	58 (4.2%)	21 (3.1%)	19 (5.6%)	​	25 (6.1%)	7 (7.8%)	9 (12.7%)	​
Other/unknown	276 (19.8%)	165 (24.2%)	81 (24.0%)	​	46 (11.3%)	10 (11.1%)	5 (7.0%)	​
BMI, kg/m^2^	27.9 (24.1, 33.1)	28.6 (24.4, 34.0)	28.3 (24.9, 32.5)	0.119	27.8 (23.1, 33.8)	27.6 (23.7, 34.0)	26.1 (24.0, 33.1)	0.962
Weight, kg	80.0 (68.0, 96.8)	85.0 (69.6, 99.2)	82.7 (69.9, 95.5)	0.037	79.0 (63.0, 96.7)	84.2 (68.1, 97.9)	77.3 (68.5, 93.4)	0.501
Comorbidities, n (%)	​	​	​	​	​	​	​	​
Chronic kidney disease, stage ≥3	394 (28.3%)	176 (25.8%)	107 (31.7%)	0.137	82 (20.1%)	31 (34.4%)	22 (31.0%)	0.005
Chronic liver disease	200 (14.3%)	115 (16.8%)	27 (8.0%)	<0.001	27 (6.6%)	7 (7.8%)	5 (7.0%)	0.926
Congestive heart failure	584 (41.9%)	241 (35.3%)	191 (56.5%)	<0.001	78 (19.2%)	20 (22.2%)	13 (18.3%)	0.772
Diabetes mellitus	530 (38.0%)	220 (32.2%)	119 (35.2%)	0.033	148 (36.4%)	36 (40.0%)	22 (31.0%)	0.496
Severity at T_0_	​	​	​	​	​	​	​	​
mSOFA-5	7.0 (4.0, 9.0)	8.0 (5.0, 10.0)	6.0 (3.0, 8.8)	<0.001	5.0 (3.0, 7.0)	6.0 (4.0, 8.0)	5.0 (3.0, 7.5)	0.008
SpO_2_/FiO_2_ ratio	194.0 (135.3, 245.0)	173.6 (123.8, 220.0)	135.7 (100.0, 196.0)	<0.001	232.5 (140.7, 442.9)	166.7 (100.1, 298.4)	125.3 (100.0, 200.0)	<0.001
Mechanical ventilation, n (%)	1,232 (88.4%)	626 (91.7%)	333 (98.5%)	<0.001	103 (25.3%)	24 (26.7%)	32 (45.1%)	0.003
Organ dysfunction to T_0_, h	7.0 (3.0, 20.0)	9.0 (4.0, 29.0)	6.0 (3.0, 17.8)	<0.001	5.0 (1.0, 14.0)	7.0 (1.0, 18.8)	2.0 (0.0, 16.5)	0.067
Organ dysfunction to suspected infection, h	1.7 (0.3, 4.4)	1.6 (0.5, 4.1)	1.8 (−1.6, 7.3)	0.927	−0.5 (−3.5, 3.4)	−0.9 (−4.6, 2.8)	0.8 (−5.0, 5.8)	0.426
Hemodynamics at shock onset (T_0_)	​	​	​	​	​	​	​	​
MAP, mmHg	59.0 (54.0, 62.0)	59.0 (54.0, 63.0)	58.0 (52.0, 62.0)	0.002	56.0 (48.5, 61.0)	54.5 (48.2, 60.0)	53.0 (41.0, 58.0)	0.044
Lactate, mmol/L	3.00 (2.40, 4.40)	3.60 (2.70, 5.90)	4.80 (3.30, 7.28)	<0.001	3.80 (2.65, 6.10)	4.70 (3.10, 8.82)	5.10 (3.25, 9.20)	<0.001
NEE, µg/kg/min	0.150 (0.080, 0.280)	0.380 (0.240, 0.545)	0.400 (0.183, 0.641)	<0.001	0.131 (0.071, 0.273)	0.373 (0.245, 0.568)	0.287 (0.158, 0.543)	<0.001
Hemodynamics, 6 h pre-T_0_	​	​	​	​	​	​	​	​
MAP (mean), mmHg	70.2 (64.8, 76.0)	70.8 (66.0, 76.2)	71.0 (66.9, 75.7)	0.147	60.4 (55.0, 67.0)	61.0 (54.4, 65.9)	61.9 (51.8, 64.8)	0.663
MAP (min), mmHg	63.0 (56.0, 68.0)	64.9 (57.0, 68.0)	65.0 (60.1, 69.0)	<0.001	52.6 (45.0, 59.0)	52.1 (43.0, 59.8)	51.6 (41.5, 59.0)	0.740
Lactate (mean), mmol/L	3.00 (2.30, 4.26)	3.30 (2.40, 5.44)	3.77 (2.17, 6.36)	<0.001	4.20 (2.76, 6.60)	4.85 (3.03, 8.51)	4.73 (2.79, 9.62)	0.080
Lactate (max), mmol/L	3.30 (2.46, 4.80)	3.70 (2.60, 6.10)	4.70 (3.10, 7.63)	<0.001	4.40 (2.95, 6.84)	4.94 (3.35, 8.63)	5.20 (3.40, 9.66)	0.011
NEE (mean), µg/kg/min	0.043 (0.000, 0.128)	0.185 (0.050, 0.344)	0.050 (0.000, 0.184)	<0.001	0.018 (0.000, 0.079)	0.099 (0.000, 0.268)	0.031 (0.000, 0.135)	<0.001
NEE (max), µg/kg/min	0.100 (0.000, 0.250)	0.342 (0.160, 0.515)	0.185 (0.000, 0.500)	<0.001	0.062 (0.000, 0.191)	0.241 (0.000, 0.492)	0.144 (0.000, 0.314)	<0.001
Treatment context	​	​	​	​	​	​	​	​
Initial antibiotic class, n (%)	​	​	​	<0.001	​	​	​	0.332
Vancomycin	547 (39.2%)	246 (36.0%)	181 (53.6%)	​	142 (34.9%)	34 (37.8%)	31 (43.7%)	​
Piperacillin-tazobactam	213 (15.3%)	116 (17.0%)	41 (12.1%)	​	119 (29.2%)	18 (20.0%)	19 (26.8%)	​
Levofloxacin	24 (1.7%)	7 (1.0%)	2 (0.6%)	​	36 (8.8%)	8 (8.9%)	2 (2.8%)	​
Ceftriaxone	126 (9.0%)	54 (7.9%)	17 (5.0%)	​	34 (8.4%)	6 (6.7%)	5 (7.0%)	​
Other/unknown	484 (34.7%)	260 (38.1%)	97 (28.7%)	​	76 (18.7%)	24 (26.7%)	14 (19.7%)	​

Continuous variables are presented as the median (IQR); categorical variables are presented as n (%). *p*-values from Kruskal–Wallis tests for continuous variables and chi-squared tests for categorical variables. Cohort sizes refer to patients retained after propensity trimming (estimated propensity ∈ [0.01, 0.99]). Mechanical-ventilation status was less completely captured in eICU-CRD than in MIMIC-IV, and the between-database comparison of this variable should be interpreted with caution.

BMI, body mass index; eICU-CRD, eICU Collaborative Research Database; IQR, interquartile range; MAP, mean arterial pressure; MIMIC-IV, Medical Information Mart for Intensive Care IV; mSOFA-5, modified five-component Sequential Organ Failure Assessment score (cardiovascular component omitted); NEE, norepinephrine-equivalent dose; S1, norepinephrine alone; S2, norepinephrine plus vasopressin; S3, norepinephrine plus epinephrine; SpO_2_/FiO_2_, peripheral oxygen saturation to fraction of inspired oxygen ratio; T_0_, time zero of septic-shock onset.

### Heterogeneity and policy learning

Five prespecified subgroups—NEE at T_0_ (<0.25 vs. ≥0.25 μg/kg/min), lactate at T_0_ (<4 vs. ≥4 mmol/L), age (<65 vs. ≥65 years), chronic kidney disease, and chronic liver disease—were analyzed by computing the same DR contrasts within each level. The NEE <0.25 stratum was the prespecified primary anchor, aligned with the SSC threshold for adjunctive vasopressin (Evans et al., 2021). To provide a structural summary of the estimated heterogeneity, a depth-2 policy tree was fit to the DR-score matrix using the policytree R package, with min.node.size set to 5% of the trimmed sample. Honest policy values for the tree, each “always-S” rule, and the observed clinician policy were obtained by fivefold cross-fitting; bootstrap stability across 2,000 resamples and implementation details are given in Supplementary eMethods S6.

### External replication and sensitivity

The full pipeline was rerun in eICU-CRD with no parameter changes; nuisance-model diagnostics for eICU-CRD are shown in Supplementary eFigure S5. Six prespecified sensitivity analyses were used to test robustness: stricter trimming (π ∈ [0.05, 0.95]); no trimming; gradient-boosted outcome model; widened strategy window of [T_0_, T_0_+6 h]; addition of a fourth phenylephrine-only arm; and Rubin-pooled MICE imputation. Three further analyses were added during revision: a landmark analysis counting follow-up from T_0_+3 h—such that deaths within the assignment window are no longer assigned by default to S1—to address immortal-time bias; reclassification of hospice discharges as deaths; and re-estimation of the primary anchor at alternative NEE thresholds (<0.20 and <0.30 μg/kg/min). Sensitivity to unmeasured confounding for the primary anchor effect was quantified by E-values for the point estimate and the confidence-interval bound nearest the null on the risk-ratio scale (VanderWeele and Ding, 2017). Sensitivity specifications and the E-value formula are given in Supplementary eMethods S7. Analyses used Python 3.11, R 4.3 (policytree), and PostgreSQL.

## Results

### Cohort

Norepinephrine alone was the predominant strategy and epinephrine the least common escalation in both cohorts. Of 94,458 ICU stays screened in MIMIC-IV, 4,183 met Sepsis-3 septic-shock criteria, 3,035 received NE within the strategy-assignment window, and 2,415 remained after propensity trimming (Figure 1). The trimmed cohort comprised 1,394 (57.7%) NE-only patients (S1), 683 (28.3%) NE plus vasopressin (S2), and 338 (14.0%) NE plus epinephrine (S3). The eICU-CRD pipeline yielded 686 shock cases, 606 NE-anchored patients, and 568 trimmed patients (S1 = 407, S2 = 90, S3 = 71).

Median age was 68 years in S1, 65 in S2, and 69 in S3 (*p* < 0.001). Severity at T_0_ increased from S1 to S3: median lactate 3.00, 3.60, and 4.80 mmol/L (*p* < 0.001) and median NEE 0.150, 0.380, and 0.400 μg/kg/min (*p* < 0.001). Pre-existing congestive heart failure was most prevalent in S3 (56.5% vs. 41.9% and 35.3%; *p* < 0.001), whereas chronic liver disease was least prevalent in S3 (8.0% vs. 14.3% and 16.8%; *p* < 0.001). Median corrected mSOFA-5 at T_0_ differed across strategies (7 in S1, 8 in S2, and 6 in S3; *p* < 0.001). Full baseline characteristics for both databases are shown in Table 1. Unadjusted in-hospital mortality was 38.1% in S1, 53.8% in S2, 44.7% in S3, and 20.6% in censored patients; corresponding 28-day renal-replacement-therapy rates were 20.3%, 36.1%, 33.9%, and 14.1%, respectively (Supplementary eTable S1).

### Average treatment effects

At the cohort level, neither escalation altered mortality on average, apart from a borderline reduction with epinephrine in the derivation cohort. In MIMIC-IV the doubly robust contrast for S2 *versus* S1 was +1.60 percentage points (pp) (95% confidence interval [CI] −7.79 to +5.47, *p* = 0.710), and for S3 *versus* S1 was −9.66 pp (95% CI −19.36 to −0.11, *p* = 0.048) (Table 2). Rubin-pooled MICE estimates were +2.73 pp (95% CI −4.69 to +10.15, *p* = 0.443) and −10.61 pp (95% CI −19.67 to −1.54, *p* = 0.015), with fractions of missing information of 0.03 and 0.05. In eICU-CRD, the corresponding bootstrap estimates were +6.36 pp (95% CI −30.19 to +25.20, *p* = 0.992) and +0.73 pp (95% CI −26.10 to +28.44, *p* = 0.961). The S2-versus-S1 contrast is the one most exposed to confounding by indication because vasopressin is typically added to patients already escalating on norepinephrine; the higher baseline norepinephrine-equivalent dose and lactate of the S2 group (Table 1) are consistent with this.

**TABLE 2:** Cohort-level average treatment effects for vasopressor-escalation strategies.

Database	Contrast	Bootstrap point estimate, pp	95% CI	*p*	MICE point estimate, pp (95% CI)	*p* [FMI]
MIMIC-IV	S2 vs. S1	+1.60	(−7.79 to +5.47)	0.710	+2.73 (−4.69 to +10.15)	0.443 [0.03]
MIMIC-IV	S3 vs. S1	−9.66	(−19.36 to −0.11)	0.048	−10.61 (−19.67 to −1.54)	0.015 [0.05]
eICU-CRD	S2 vs. S1	+6.36	(−30.19 to +25.20)	0.992	Not estimated	​
eICU-CRD	S3 vs. S1	+0.73	(−26.10 to +28.44)	0.961	Not estimated	​

Average treatment effects on in-hospital mortality (percentage points, pp; negative values favor the adjunct strategy). Primary estimates are cross-fitted doubly robust estimates, with 95% CIs and *p*-values from 2,000 patient-level bias-corrected and accelerated (BCa) bootstrap resamples (random seed 42). The MICE point estimate and *p* [FMI] columns report a missing-data sensitivity analysis (multiple imputation, m = 5 imputations, seeds 42–46) pooled by Rubin’s rules, available for MIMIC-IV only; eICU-CRD effects were estimated at the doubly robust score level, for which patient-level imputation is not applicable.

BCa, bias-corrected and accelerated; CI, confidence interval; eICU-CRD, eICU Collaborative Research Database; FMI, fraction of missing information; MICE, multiple imputation by chained equations; MIMIC-IV, Medical Information Mart for Intensive Care IV; pp, percentage points; S1, norepinephrine alone; S2, norepinephrine plus vasopressin; S3, norepinephrine plus epinephrine.

### Subgroup heterogeneity

**FIGURE 2:**
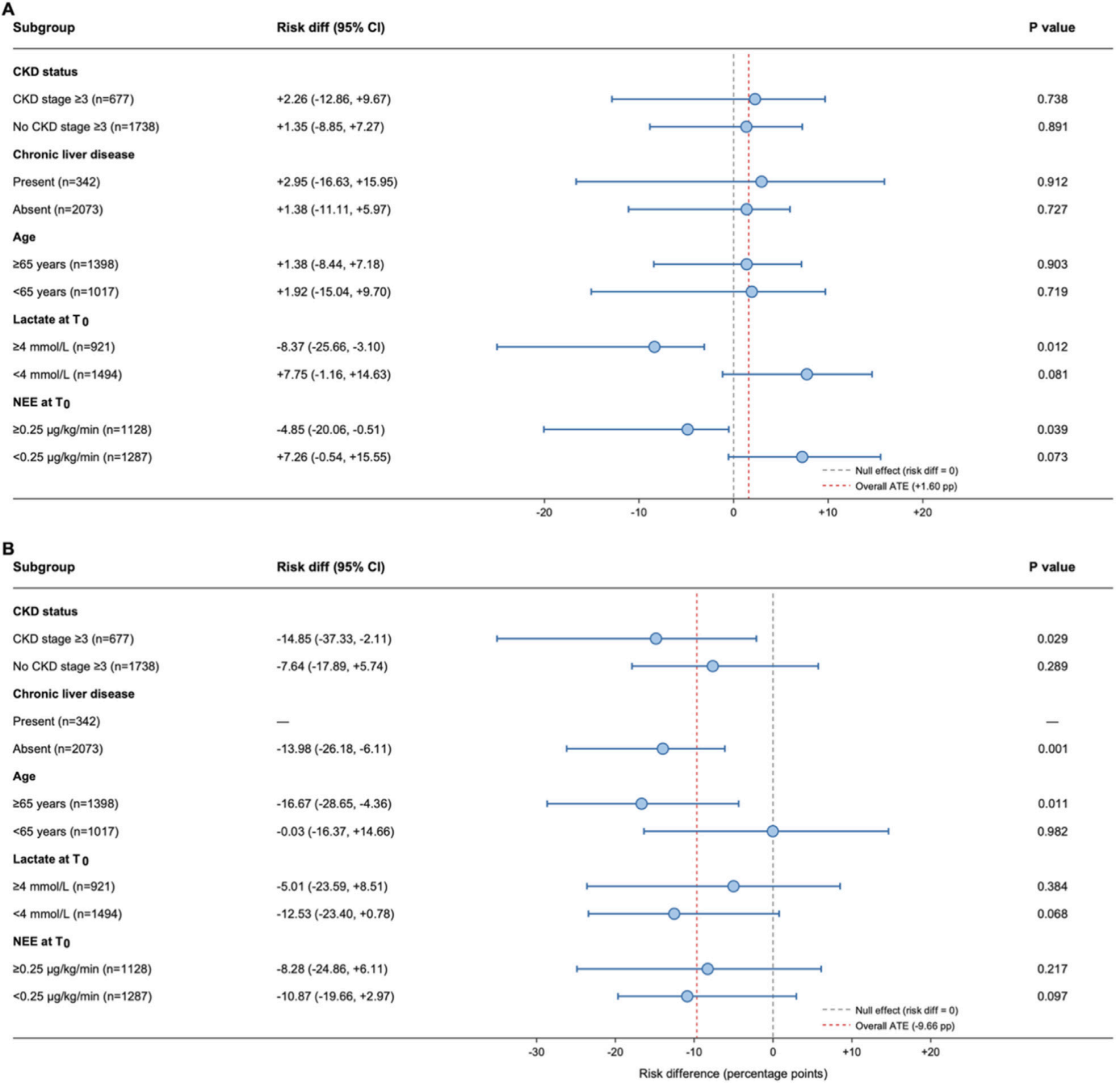
Subgroup heterogeneity of vasopressor-strategy effects on in-hospital mortality in MIMIC-IV. Forest plots of doubly robust risk differences within five prespecified subgroups, shown for the S2 *versus* S1 contrast in **(A)** and the S3 *versus* S1 contrast in **(B)**. Each row reports the subgroup level, stratum size, point estimate, 95% CI, and *p*-value; point estimates and CIs are from 2,000 patient-level bias-corrected and accelerated (BCa) bootstrap resamples (random seed 42) of the cross-fitted doubly robust learner. The gray dashed line marks the null effect (risk difference = 0 pp) and the red dashed line marks the cohort-level overall ATE for that contrast (+1.60 pp in **(A)**; −9.66 pp in **(B)**). Effects are expressed in percentage points (pp): positive values indicate higher mortality *versus* the reference S1, and negative values indicate lower mortality *versus* S1. For **(B)**, the chronic-liver-disease “Present” stratum is shown as not estimable because the S3 cell within this stratum contained 27 patients. ATE, average treatment effect; CI, confidence interval; CKD, chronic kidney disease; MIMIC-IV, Medical Information Mart for Intensive Care IV; NEE, norepinephrine-equivalent dose; pp, percentage points; S1, norepinephrine alone; S2, norepinephrine plus vasopressin; S3, norepinephrine plus epinephrine; T_0_, time zero of septic-shock onset.

Any epinephrine benefit was concentrated in lower-severity strata, and only one subgroup survived multiplicity correction. Subgroup contrasts are shown in Figure 2 and summarized in Table 3, with the long-format estimates and per-strategy cell sizes in Supplementary eTables S2, S3. The S3-versus-S1 point estimate was negative in most strata. Among these stratifications, it reached nominal significance only in older patients: −10.87 pp at NEE <0.25 μg/kg/min (n = 1,287; 95% CI −19.66 to +2.97, *p* = 0.097) *versus* −8.28 pp at NEE ≥0.25 (n = 1,128; *p* = 0.217) and −12.53 pp at lactate <4 mmol/L (n = 1,494; 95% CI −23.40 to +0.78, *p* = 0.068) *versus* −5.01 pp at lactate ≥4 (n = 921; *p* = 0.384). The age stratification yielded −16.67 pp at age ≥65 (n = 1,398; 95% CI −28.65 to −4.36, *p* = 0.011) and −0.03 pp at age <65 (n = 1,017; *p* = 0.982). The S2-versus-S1 contrast was directionally positive but non-significant in the lower-severity strata (lactate <4: +7.75 pp, 95% CI −1.16 to +14.63, *p* = 0.081; NEE <0.25: +7.26 pp, 95% CI −0.54 to +15.55, *p* = 0.073) and negative in the higher-severity strata (lactate ≥4: −8.37 pp, 95% CI −25.66 to −3.10, *p* = 0.012; NEE ≥0.25: −4.85 pp, 95% CI −20.06 to −0.51, *p* = 0.039). Among comorbidity strata, the S3-versus-S1 reduction was significant in the chronic-liver-absent stratum (−13.98 pp, 95% CI −26.18 to −6.11, *p* = 0.001) and the chronic-kidney-disease stratum (−14.85 pp, 95% CI −37.33 to −2.11, *p* = 0.029), whereas the S2-versus-S1 contrast was null throughout; the chronic-liver-present stratum S3-versus-S1 contrast was not estimable due to an S3 cell size of 27. After Bonferroni correction at the prespecified threshold of 0.006 for the family of secondary tests, only the chronic-liver-absent S3-versus-S1 reduction (*p* = 0.001) remained significant.

**TABLE 3:** Subgroup heterogeneity of average treatment effects in MIMIC-IV.

Subgroup	Contrast	Stratum 1			Stratum 2		
		Level (n)	ATE, pp (95% CI)	*p*	Level (n)	ATE, pp (95% CI)	*p*
NEE at T_0_, µg/kg/min	S3 vs. S1	<0.25 (1,287)	−10.87 (−19.66 to +2.97)	0.097	≥0.25 (1,128)	−8.28 (−24.86 to +6.11)	0.217
	S2 vs. S1	<0.25 (1,287)	+7.26 (−0.54 to +15.55)	0.073	≥0.25 (1,128)	−4.85 (−20.06 to −0.51)	0.039
Lactate at T_0_, mmol/L	S3 vs. S1	<4 (1,494)	−12.53 (−23.40 to +0.78)	0.068	≥4 (921)	−5.01 (−23.59 to +8.51)	0.384
	S2 vs. S1	<4 (1,494)	+7.75 (−1.16 to +14.63)	0.081	≥4 (921)	−8.37 (−25.66 to −3.10)	0.012
Age, years	S3 vs. S1	<65 (1,017)	−0.03 (−16.37 to +14.66)	0.982	≥65 (1,398)	−16.67 (−28.65 to −4.36)	0.011
	S2 vs. S1	<65 (1,017)	+1.92 (−15.04 to +9.70)	0.719	≥65 (1,398)	+1.38 (−8.44 to +7.18)	0.903
Chronic kidney disease, stage ≥3	S3 vs. S1	No (1,738)	−7.64 (−17.89 to +5.74)	0.289	Yes (677)	−14.85 (−37.33 to −2.11)	0.029
	S2 vs. S1	No (1,738)	+1.35 (−8.85 to +7.27)	0.891	Yes (677)	+2.26 (−12.86 to +9.67)	0.738
Chronic liver disease	S3 vs. S1	No (2,073)	−13.98 (−26.18 to −6.11)	0.001	Yes (342)	Not estimable	​
	S2 vs. S1	No (2,073)	+1.38 (−11.11 to +5.97)	0.727	Yes (342)	+2.95 (−16.63 to +15.95)	0.912

Heterogeneity of treatment effects across five prespecified subgroups in MIMIC-IV. Effects expressed in percentage points (pp) as risk differences for in-hospital mortality, with 95% CIs and p-values from 2,000 patient-level bias-corrected and accelerated (BCa) bootstrap resamples (random seed 42). Positive values indicate higher mortality *versus* the reference S1; negative values indicate lower mortality *versus* S1. The S3 *versus* S1 contrast was not estimable in the chronic-liver-disease present (Yes) stratum because the corresponding S3 cell contained 27 patients.

ATE, average treatment effect; CI, confidence interval; CKD, chronic kidney disease; MIMIC-IV, Medical Information Mart for Intensive Care IV; NEE, norepinephrine-equivalent dose; pp, percentage points; S1, norepinephrine alone; S2, norepinephrine plus vasopressin; S3, norepinephrine plus epinephrine; T_0_, time zero of septic-shock onset.

### Policy learning

The data-derived tree split first on baseline severity rather than on any demographic variable, echoing the prespecified dose anchor. The depth-2 policy tree fit to the doubly robust scores is shown in Supplementary eFigure S6. The first split was on peak norepinephrine-equivalent dose during the 6 h preceding T_0_ (≤ 0.16 μg/kg/min). Within the lower-dose branch, age ≤60 years was assigned NE-only (n = 401, 16.6%) and age >60 years was assigned NE + epinephrine (n = 792, 32.8%); within the higher-dose branch, MAP at T_0_ ≤ 57 mmHg was assigned NE + vasopressin (n = 440, 18.2%) and MAP >57 mmHg was assigned NE + epinephrine (n = 782, 32.4%).

**FIGURE 3:**
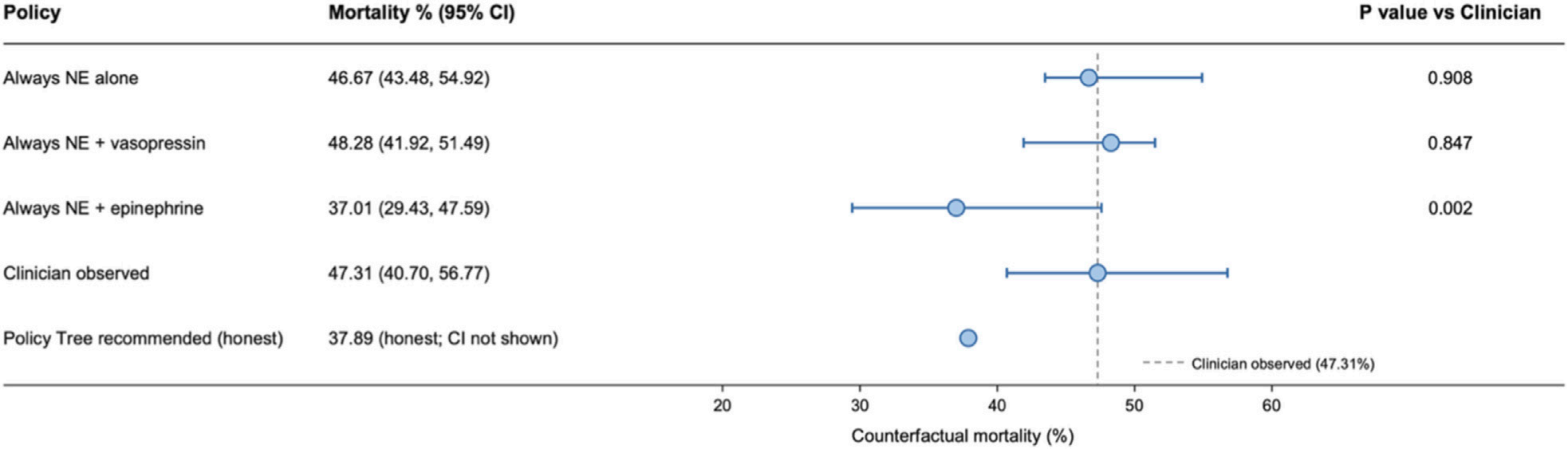
Honest cross-fit counterfactual mortality of fixed-strategy and learned policies in MIMIC-IV. Forest plot of the counterfactual in-hospital mortality for each candidate policy in the trimmed MIMIC-IV cohort (n = 2,415), estimated by fivefold honest cross-fitting of the doubly robust scores. For the three fixed strategies (always NE alone, always NE + vasopressin, always NE + epinephrine) and the clinician-observed policy, each row reports the point estimate, 95% CI, and the *p*-value comparing that policy against the clinician-observed policy; point estimates and confidence intervals are from 2,000 patient-level bootstrap resamples (random seed 42) with bias-corrected and accelerated (BCa) intervals. The learned policy-tree value is shown as a point estimate only: because the tree is data-adaptively learned, a bootstrap interval holding the learned rule fixed would be anticonservative, so no confidence interval or p-value is displayed for this row; the in-sample (non-honest) policy-tree value and its optimism gap relative to the honest estimate are reported in Supplementary eTable S4 instead. The gray dashed vertical line marks the clinician-observed counterfactual mortality (47.31%) as the reference; the statistical comparison of each policy against clinician care is given by the p-value in the right-hand column. Because each policy value and the clinician-observed value are estimated in the same cohort and are therefore correlated, a policy’s own confidence interval may overlap the reference line even when the paired difference is significant. The depth-2 policy tree was fit using the policytree R package, with min.node.size set to 5% of the trimmed sample; its structure is shown in Supplementary eFigure S6 and is intended as a descriptive summary of estimated heterogeneity rather than as a clinical decision rule. CI, confidence interval; MIMIC-IV, Medical Information Mart for Intensive Care IV; NE, norepinephrine.

Honest fivefold cross-fit policy values are shown in Figure 3 and Table 4. The clinician-observed policy yielded a counterfactual mortality of 47.31% (95% CI 40.70–56.77). Always-S3 was 37.01% (95% CI 29.43 to 47.59, *p* = 0.002 *versus* clinician); always-S1 was 46.67% (43.48–54.92, *p* = 0.908); and always-S2 was 48.28% (41.92–51.49, *p* = 0.847). The policy-tree assignment was 37.89% (honest fivefold; confidence interval not estimated). The in-sample (non-honest) policy-tree value was 27.87% (95% CI 18.73–32.34), corresponding to an optimism gap of 10.02 pp relative to the cross-fit estimate (Supplementary eTable S4).

**TABLE 4:** Honest cross-fit counterfactual mortality of fixed-strategy and learned policies (MIMIC-IV).

Policy	Counterfactual mortality, %	SE	95% CI	*p* vs. clinician
Clinician (observed policy)	47.31	4.00	(40.70–56.77)	—
Always S1 (NE alone)	46.67	2.69	(43.48–54.92)	0.908
Always S2 (NE + vasopressin)	48.28	2.31	(41.92–51.49)	0.847
Always S3 (NE + epinephrine)	37.01	4.45	(29.43–47.59)	0.002
Policy tree recommended (honest)	37.89	—	not shown	—

Counterfactual in-hospital mortality of each candidate policy in the MIMIC-IV trimmed cohort, estimated by fivefold honest cross-fitting of the doubly robust scores. 95% CIs and p-values from 2,000 patient-level bootstrap resamples (random seed 42) with bias-corrected and accelerated (BCa) intervals; p-values compare each policy against the clinician-observed policy. SE is the standard deviation of the 2,000 bootstrap replicates. The depth-2 policy tree was fit using the policytree R package, with min.node.size set to 5% of the trimmed sample; the learned policy-tree value is the honest cross-fit estimate, shown as a point only. Since the tree structure is data-adaptive, naive patient-level resampling refits the tree in every replicate and does not yield a valid bootstrap standard error, so SE and CI are omitted (“—”) for the honest policy-tree value. The corresponding in-sample (non-honest) policy-tree value (27.87%), its standard error (3.29 pp), and the optimism gap (+10.02 pp) are reported in Supplementary eTable S4.

CI, confidence interval; MIMIC-IV, Medical Information Mart for Intensive Care IV; NE, norepinephrine; SE, standard error; S1, norepinephrine alone; S2, norepinephrine plus vasopressin; S3, norepinephrine plus epinephrine.

### External replication

**FIGURE 4:**
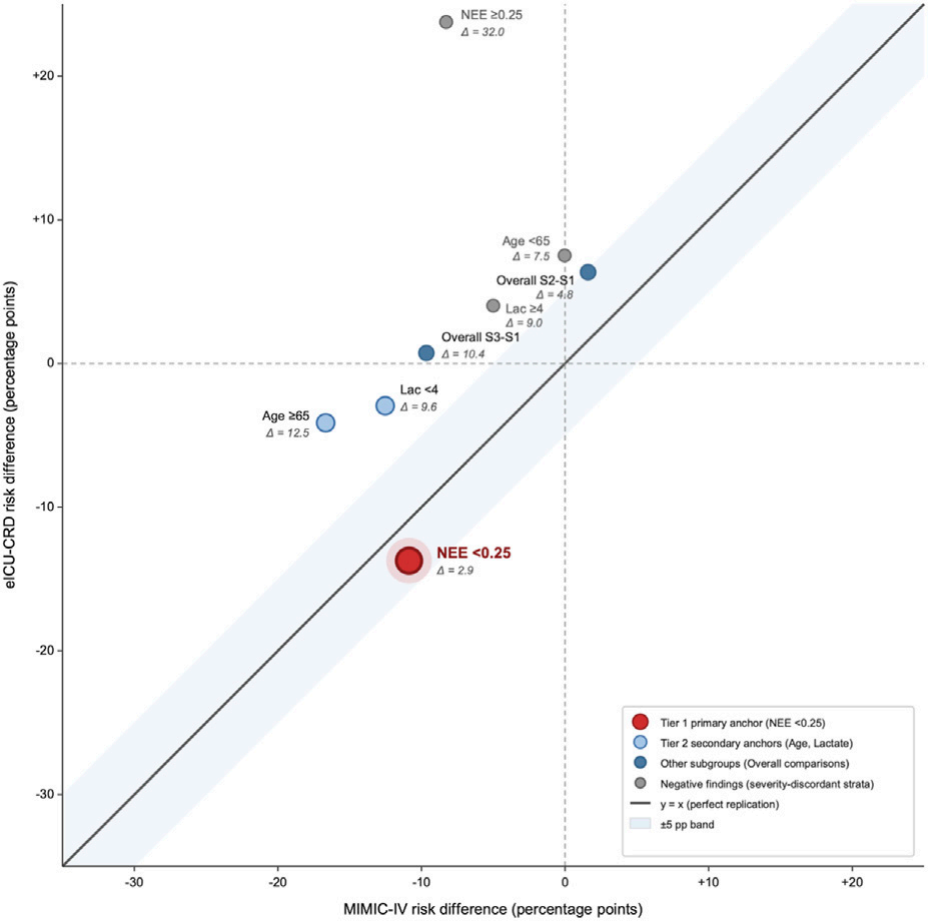
External replication of MIMIC-IV per-stratum risk differences in eICU-CRD. Scatter plot of doubly robust risk differences (S3 vs. S1, except where indicated) for each subgroup, plotted with the MIMIC-IV estimate on the x-axis and the eICU-CRD estimate on the y-axis. Each point is annotated with the subgroup name and the absolute difference Δ between the two cohort estimates. The diagonal black line (y = x) marks perfect replication; the shaded blue band marks a ±5 pp tolerance around perfect replication; the gray dashed lines mark the null effect on each axis. Points are colored by replication tier: red, the Tier 1 prespecified primary anchor (NEE <0.25 μg/kg/min); light blue, the Tier 2 prespecified secondary anchors (age ≥65 years and lactate <4 mmol/L); dark blue, the cohort-level overall S2 *versus* S1 and S3 *versus* S1 contrasts; and gray, the complementary, non-hypothesized strata that were not prespecified for replication and serve as negative controls (NEE ≥0.25, lactate ≥4, age <65). The Tier 1 anchor lies within the ±5 pp band, with both cohorts indicating lower mortality under S3 than S1; the cohort-level S3 *versus* S1 contrast reverses sign in eICU-CRD. Numerical estimates and 95% CIs are reported in Table 5. CI, confidence interval; eICU-CRD, eICU Collaborative Research Database; MIMIC-IV, Medical Information Mart for Intensive Care IV; NEE, norepinephrine-equivalent dose; pp, percentage points; S1, norepinephrine alone; S2, norepinephrine plus vasopressin; S3, norepinephrine plus epinephrine; Δ, absolute difference between MIMIC-IV and eICU-CRD point estimates.

External replication in eICU-CRD was partial: the low-dose anchor was directionally consistent, whereas the cohort-level contrast was not. eICU-CRD *versus* MIMIC-IV per-stratum point estimates and replication tiers are shown in Figure 4 and Table 5. The Tier-1 anchor (NEE <0.25 μg/kg/min, S3 *versus* S1) was directionally consistent at −13.72 pp in eICU (MIMIC −10.87 pp; Δ = 2.85 pp; 95% CI −48.24 to +9.47, *p* = 0.204). The two Tier-2 anchors were directionally consistent: lactate <4 was −2.94 pp (Δ = 9.59) and age ≥65 was −4.13 pp (Δ = 12.54). The Tier-3 overall S2-versus-S1 contrast was concordant positive (MIMIC +1.60, eICU +6.36; Δ = 4.76), and the overall S3-versus-S1 contrast reversed in eICU (MIMIC −9.66, eICU +0.73). Every eICU-CRD interval was wide—the anchor alone spanned −48.24 to +9.47 pp—thus, these external estimates establish direction but are individually too imprecise to read as point confirmation; the smaller secondary strata are reported as directional checks rather than independent tests.

**TABLE 5:** External replication of MIMIC-IV findings in the eICU-CRD validation cohort.

Tier	Subgroup	Contrast	n (MIMIC/eICU)	MIMIC-IV		eICU-CRD		Δ, pp	Replication
				ATE, pp (95% CI)	*p*	ATE, pp (95% CI)	*p*		
Tier 1	NEE <0.25 μg/kg/min	S3 vs. S1	1,287/349	−10.87 (−19.66 to +2.97)	0.097	−13.72 (−48.24 to +9.47)	0.204	2.85	Directionally consistent
Tier 2	Age ≥65 years	S3 vs. S1	1,398/331	−16.67 (−28.65 to −4.36)	0.011	−4.13 (−38.00 to +25.89)	0.792	12.54	Wide attenuation
Tier 2	Lactate <4 mmol/L	S3 vs. S1	1,494/269	−12.53 (−23.40 to +0.78)	0.068	−2.94 (−22.33 to +42.37)	0.909	9.59	Directionally consistent
Tier 3	Overall (full cohort)	S2 vs. S1	2,415/568	+1.60 (−7.79 to +5.47)	0.710	+6.36 (−30.19 to +25.20)	0.992	4.76	Concordant null
Tier 3	Overall (full cohort)	S3 vs. S1	2,415/568	−9.66 (−19.36 to −0.11)	0.048	+0.73 (−26.10 to +28.44)	0.961	10.39	Direction reversal
Negative	NEE ≥0.25 μg/kg/min	S3 vs. S1	1,128/219	−8.28 (−24.86 to +6.11)	0.217	+23.76 (−19.44 to +90.52)	0.262	32.04	Direction reversal
Negative	Lactate ≥4 mmol/L	S3 vs. S1	921/299	−5.01 (−23.59 to +8.51)	0.384	+4.03 (−44.09 to +44.20)	0.969	9.04	Direction reversal
Negative	Age <65 years	S3 vs. S1	1,017/237	−0.03 (−16.37 to +14.66)	0.982	+7.52 (−33.56 to +63.87)	0.730	7.55	Concordant null

External replication of subgroup and cohort-level effects from MIMIC-IV in eICU-CRD. Effects expressed in percentage points (pp) as risk differences for in-hospital mortality, with 95% CIs and *p*-values from 2,000 patient-level bias-corrected and accelerated (BCa) bootstrap resamples (random seed 42) in each cohort separately. Δ is the absolute difference between MIMIC-IV and eICU-CRD point estimates. Tier 1 is the prespecified primary anchor; Tier 2 are the prespecified secondary anchors; Tier 3 is the cohort-level reference; the “Negative” rows are the high-severity complementary strata where no replication was prespecified. Positive values indicate higher mortality *versus* the reference S1; negative values indicate lower mortality *versus* S1.

ATE, average treatment effect; CI, confidence interval; eICU-CRD, eICU Collaborative Research Database; MIMIC-IV, Medical Information Mart for Intensive Care IV; NEE, norepinephrine-equivalent dose; pp, percentage points; S1, norepinephrine alone; S2, norepinephrine plus vasopressin; S3, norepinephrine plus epinephrine.

### Sensitivity analyses and unmeasured confounding

The anchor estimate stayed negative across every sensitivity specification and proved only moderately sensitive to unmeasured confounding. Six prespecified sensitivity specifications are summarized in Supplementary eFigure S7 and Supplementary eTable S5. The NEE <0.25 anchor effect remained negative under every specification, ranging from −5.32 pp (6-h strategy window) to −18.96 pp (untrimmed). The Rubin-pooled MICE estimate was −11.45 pp for the anchor, within this range. Three analyses were added in revision. Varying the anchor threshold from <0.20 to <0.30 μg/kg/min left the low-dose S3-versus-S1 estimate stable between −10.9 and −12.0 pp, negative throughout and directionally concordant in eICU-CRD. A landmark analysis counting follow-up from T_0_+3 h attenuated but did not reverse the contrast (overall S3 vs. S1 −8.68 pp, 95% CI −18.97 to +1.18; anchor −8.80 pp), indicating that immortal-time bias does not account for the signal. Reclassifying hospice discharges as deaths strengthened the anchor contrast to −14.69 pp (95% CI −24.18 to −1.17, *p* = 0.047). Bootstrap risk-difference distributions for the four overall contrasts are in Supplementary eFigure S8. E-values for the MIMIC-IV NEE <0.25 anchor were 2.19 for the point estimate and 1.00 for the confidence-interval bound nearest the null; for the age ≥65 anchor, they were 2.60 and 1.46 (Supplementary eTable S6). An unmeasured confounder would need to be associated with both epinephrine use and mortality at a risk ratio near 2.2 to move the anchor point estimate to the null, a strength greater than that of any measured predictor in these data. The interval is more fragile: its bound nearest the null corresponds to an E-value of 1.0; hence, essentially no unmeasured confounding is required to reach it. Thus, the point estimate is moderately robust to unmeasured confounding, whereas the interval is not.

## Discussion

In two intensive care databases, escalating from norepinephrine alone to norepinephrine plus epinephrine produced no cohort-level mortality difference that reached statistical significance, and adding vasopressin within the early-resuscitation window likewise showed no significant difference from norepinephrine alone in either cohort. The one contrast that was directionally consistent across both databases lay within a prespecified severity stratum: among patients with a norepinephrine-equivalent dose below 0.25 μg/kg/min at time zero, the doubly robust estimate for norepinephrine plus epinephrine *versus* norepinephrine alone was about 11 percentage points lower in MIMIC-IV and, over a wide interval, 14 points lower in eICU-CRD; in MIMIC-IV the E-value exceeded the strength of unmeasured confounding that would be needed to explain the estimate away. We read these results as hypothesis-generating evidence of severity-dependent heterogeneity, and not as support for a uniform benefit of any single escalation strategy.

The pattern is broadly compatible with the prior randomized and observational record. The CATS trial, the largest randomized comparison of epinephrine *versus* norepinephrine plus dobutamine in septic shock, reported 28-day mortality of 40% *versus* 34% (p = 0.31) and no difference in safety, leaving the comparative position of epinephrine in this population unsettled rather than refuted (Annane et al., 2007). The VANISH trial reported no kidney-failure-free-day benefit from early vasopressin and an effect estimate that did not exclude clinically important effects in either direction (Gordon et al., 2016); a secondary analysis of VASST suggested vasopressin benefit in patients with less severe shock that has not been confirmed in subsequent randomized work (Russell et al., 2008); and recent observational studies have supported earlier vasopressin initiation in selected populations, including a target trial emulation by White and colleagues that reported effect modification by norepinephrine-equivalent dose (White et al., 2025) and a recent network meta-analysis of randomized trials in which adjunctive vasopressin did not rank above norepinephrine alone for 28-day mortality (Jia et al., 2023). A narrative review of vasoactive timing concluded that early vasopressor initiation within the first 6 h of shock onset is associated with lower mortality but emphasized the residual uncertainty around adjunctive escalation (Ammar et al., 2022). A 2025 meta-analysis of timing studies again reported a pooled relative risk of 0.84 (95% CI 0.71–0.99) for early *versus* late initiation (Bhattacharjee et al., 2025), whereas the trial sequential analysis flagged the underlying evidence as low certainty. Our anchor finding—a signal concentrated below the SSC dose threshold—fits this literature, which on balance suggests that any vasopressin or epinephrine effect, if it exists, is unlikely to be uniform across the septic-shock population.

Early adjunctive vasopressin showed no significant mortality difference from norepinephrine alone in MIMIC-IV (+1.60 pp; 95% CI −7.79 to +5.47; *p* = 0.710), in keeping with the neutral primary results of VASST (Russell et al., 2008) and VANISH (Gordon et al., 2016). We read this null result cautiously, for three reasons. The first is confounding by indication, the dominant threat to any observational vasopressin estimate: in current practice vasopressin is most often added when a patient is deteriorating despite ongoing norepinephrine titration, a pattern evident here in the higher norepinephrine-equivalent and pre-T_0_ peak doses of the S2 group. The Cleveland Clinic cohort showed that each higher quartile of norepinephrine-equivalent dose and lactate at the time of vasopressin initiation was independently associated with higher in-hospital mortality—the time-varying severity that baseline-anchored emulation cannot fully balance (Sacha et al., 2022)—and a later synthesis argued that the apparent harm of late vasopressin reflects severity at the moment of escalation rather than the drug itself (Sacha and Bauer, 2023). Propensity weighting and the doubly robust estimator handle measured baseline confounders, but residual confounding by short-term clinical trajectory can persist, as has recently been re-emphasized for target trial emulation in observational data (Fu, 2023; Hubbard et al., 2024). The second reason is precision: the eICU-CRD estimate for the same contrast was also positive but far too wide to interpret (+6.36 pp; 95% CI −30.19 to +25.20; *p* = 0.992), reflecting the small external sample rather than any signal. The third reason is sensitivity to unmeasured confounding: the E-value for this contrast was the lowest we examined; thus, a confounder associated with both treatment and outcome at a risk ratio near 1.24 would account for the entire point estimate. We therefore read the vasopressin contrast not as evidence for or against current SSC guidance (Evans et al., 2021) but as a question that only prospective, dose-anchored randomization can settle.

The depth-2 policy tree is a structural summary of the estimated heterogeneity and not a clinical decision rule, and several features argue against any bedside reading. Its first split is on baseline norepinephrine-equivalent dose; within the low-dose group, it directs older patients toward epinephrine-containing escalation—a rule that is at odds with the safety profile of epinephrine, which in a recent network meta-analysis of randomized trials carried the highest incidence of myocardial infarction and peripheral ischemia among catecholamines (Jia et al., 2023). This concern is sharpest in the older patients with the high coronary-disease burden observed in our S3 group. The tree is also structurally unstable: across bootstrap resamples the modal first-split variable was selected in only about 20% of replicates; thus, it should not be treated as a validated rule. As it was fit to doubly robust scores derived from clinician-chosen exposures, the structure it recovers reflects severity-correlated patterns in current practice rather than a causally optimized policy. The policy-value comparisons reported earlier answer a different question from the pairwise average treatment effects and should not be set against them: a pairwise contrast estimates the effect of one agent *versus* another with the population held fixed, whereas a policy value is the expected outcome if a single rule were applied to the whole cohort. That the marginal epinephrine-*versus*-norepinephrine contrast favored epinephrine while a blanket always-epinephrine rule did not outperform—and in fact underperformed—selective clinician practice (policy value 37.01 vs. 47.31; *p* = 0.002) is not a contradiction but a direct consequence of heterogeneity. The tree is best read as a hypothesis-generating map of where treatment-response heterogeneity concentrates, principally along axes of pre-T_0_ severity, not as a recommendation about which agent to add. This framing also separates the present analysis from the two approaches that motivated it: a reinforcement-learning initiation rule optimizes a policy that is hard to inspect, and a single target-trial emulation answers one binary contrast, whereas the tree here is offered only as an interpretable map of where heterogeneity concentrates, not as a rule to be deployed.

External replication in eICU-CRD was partial. The primary anchor was directionally consistent: norepinephrine plus epinephrine *versus* norepinephrine alone was −13.72 pp among patients below the dose threshold, close in magnitude to the MIMIC-IV estimate, though over a confidence interval that was wide and crossed the null, so the external data corroborate rather than confirm. Of the two secondary strata, the lactate <4 mmol/L contrast remained negative, whereas the age ≥65 contrast attenuated sharply, from −16.67 pp in MIMIC-IV to −4.13 in eICU-CRD. The full-cohort contrast changed sign, from −9.66 pp in MIMIC-IV to +0.73 in eICU-CRD (95% CI −26.10 to +28.44; *p* = 0.961), again over an interval spanning a wide range of plausible effects.

Three explanations fit this pattern, and they are not mutually exclusive. The population-average effect may be close to null, with the MIMIC-IV signal arising from severity strata that make up a larger share of that cohort than of eICU-CRD. The discrepancy may be statistical: after trimming, the eICU-CRD sample was small (n = 568), the secondary strata smaller still, and outcome-model calibration in the S2 and S3 arms weaker. Thus, a subgroup estimate such as the age ≥65 contrast is imprecise and prone to exactly the attenuation expected when an estimate fitted in one cohort is carried to another. The difference may be substantive: the two databases differ in case-mix, vasopressor dosing and escalation practice, and the completeness and resolution of their recorded physiology, any of which can shift the balance of treated severity. We do not adjudicate among the three. None supports a uniform population-level benefit of either escalation, and all are consistent with an effect that, if real, is concentrated in defined severity strata rather than spread evenly across patients.

Two methodological points bear directly on interpretation. The norepinephrine-equivalent dose <0.25 μg/kg/min stratum was the single prespecified primary anchor, chosen on physiological and guideline-aligned grounds (Evans et al., 2021); the four secondary subgroups—lactate, age, chronic kidney disease, and chronic liver disease—were planned but not designated as primary tests. The anchor estimate did not reach conventional significance (*p* = 0.097) but stayed negative under multiple imputation (−11.45 pp) and across every sensitivity specification we ran. Among the secondary strata, only the absence of chronic liver disease (*p* = 0.001) survived a conservative Bonferroni threshold of 0.006 for the family of secondary tests; the age ≥65 contrast, though nominally significant (*p* = 0.011), did not. We do not treat strata that failed correction as confirmatory evidence of effect modification.

Several limitations qualify these results. Both databases are retrospective, and target trial emulation cannot remove confounding by indication for time-varying severity, most acutely for vasopressin co-administration (Fu, 2023; Hubbard et al., 2024). Strategy was assigned over the first 3 h after time zero while survival was counted from time zero; hence, a patient had to survive long enough to receive a second agent—an immortal-time interval that, by construction, places deaths occurring before any second agent in the norepinephrine-alone arm and can inflate its apparent mortality. Two observations bound this concern: crude in-hospital mortality was nonetheless lowest in the norepinephrine-alone arm (38.1%, against 53.8% for vasopressin and 44.7% for epinephrine), and a landmark analysis beginning follow-up at the end of the assignment window attenuated the anchor estimate only modestly, from −10.87 to −8.80 pp. The bias is present but unlikely to generate the anchor signal. Although baseline mechanical-ventilation status and the SpO_2_/FiO_2_ ratio were included as respiratory-severity measures, covariate balance holds only at time zero, and residual confounding by post-baseline trajectory cannot be excluded. Finally, window-based assignment captures the decision to add an adjunctive agent but not subsequent dose escalation or de-escalation.

These findings are not a basis for changing first-line practice, but they carry two research implications. First, trials of vasopressor escalation should randomize within severity strata rather than enroll uniform cohorts, as uniform designs may keep diluting heterogeneity that sits in defined strata; the norepinephrine-equivalent dose threshold of 0.25 μg/kg/min, used here as the prespecified anchor and aligned with SSC guidance (Evans et al., 2021), is one operational stratifier. Second, the choice of escalation strategy may belong with broader efforts to link sepsis subphenotypes—host response and clinical trajectory—to differential treatment effect (Seymour et al., 2019). Read as a map rather than a rule, the policy tree points to severity at the time of escalation, rather than any single demographic or clinical axis, as the most informative dimension on which such trials might be stratified.

In adults with septic shock receiving norepinephrine, the average mortality effect of adding epinephrine or vasopressin was not uniform. The clearest signal—and the only directionally consistent one across both databases—was lower mortality with norepinephrine plus epinephrine among patients escalating from a norepinephrine-equivalent dose below 0.25 μg/kg/min, a finding we regard as hypothesis-generating rather than established. The vasopressin contrast was null in both cohorts and, given residual confounding by indication, neither supports nor excludes a real effect. Taken together, the data describe where treatment-effect heterogeneity concentrates rather than identify a single best escalation strategy.

## Data Availability

Publicly available datasets were analyzed in this study. These data can be found at: MIMIC-IV v3.1 (https://physionet.org/content/mimiciv/3.1/) and eICU-CRD v2.0 (https://physionet.org/content/eicu-crd/2.0/), fully de-identified datasets hosted on the PhysioNet repository. Access requires completion of CITI training and acceptance of the PhysioNet Data Use Agreement.
