# Supplementary material for: Severity-stratified vasopressor-escalation strategies in septic shock: a cross-fitted doubly robust analysis with external replication
Source: Front Pharmacol. 2026 Jul 23;17:1876555. doi: 10.3389/fphar.2026.1876555 (PMC13446109; doi:10.3389/fphar.2026.1876555)
Supplement: Supplementary file 1 [file DataSheet1.docx]

**Supplementary Materials**

***Severity-stratified vasopressor escalation strategies in septic shock: a cross-fitted doubly-robust analysis with external replication***

Yuhang Zhang¹, Menglin Han², Xiaosu Liu¹, Guanyu Chen³, Lulu Zhang³*

**Affiliations:**

¹ Department of Anesthesiology, The Second Affiliated Hospital of Chongqing Medical University, Chongqing, 400010, China.

2 Department of Critical Care Medicine, The Second Affiliated Hospital of Chongqing Medical University, Chongqing, 400010, China.

3 Department of Anesthesiology, Zigong Fourth People's Hospital, Zigong, Sichuan, 643000, China.

***Corresponding author:**

Lulu Zhang

Department of Anesthesiology, Zigong Fourth People's Hospital, Zigong, Sichuan, 643000, China. Email: zhangll.anes.zfph@suse.edu.cn

**Contents:**

eMethods S1. Sepsis-3 septic shock definition and the five-component modified SOFA (mSOFA-5) score

eMethods S2. Norepinephrine-equivalent (NEE) dose conversion

eMethods S3. Baseline covariate definitions, missingness assessment and missing-at-random justification

eMethods S4. Cross-fitted doubly-robust learner : outcome and propensity model formulations

eMethods S5. Bootstrap protocol and cross-implementation numerical check

eMethods S6. Policy tree fitting, honest cross-fit policy values and bootstrap stability

eMethods S7. Prespecified sensitivity specifications and E-value formula

eTable S1. Cohort-level outcomes by treatment strategy in MIMIC-IV and eICU-CRD

eTable S2. Subgroup heterogeneity of average treatment effects in MIMIC-IV — long format

eTable S3. Per-strategy cell sizes within each prespecified subgroup level (MIMIC-IV trimmed cohort)

eTable S4. In-sample versus honest cross-fit counterfactual mortality and optimism gap for each candidate policy (MIMIC-IV)

eTable S5. Sensitivity analyses of the doubly-robust contrast estimates (MIMIC-IV)

eTable S6. E-values on the risk-ratio scale for the prespecified anchor and complementary contrasts, in MIMIC-IV and eICU-CRD

eFigure S1. Distribution of norepinephrine-equivalent dose at time zero across treatment strategies in the MIMIC-IV norepinephrine-anchored cohort

eFigure S2. Pairwise Pearson correlation matrix of continuous baseline covariates in the trimmed MIMIC-IV cohort

eFigure S3. Per-covariate missingness in the MIMIC-IV norepinephrine-anchored cohort, ordered by missing rate

eFigure S4. Nuisance-model diagnostics: propensity overlap and outcome-model calibration in MIMIC-IV

eFigure S5. Nuisance-model diagnostics in eICU-CRD: per-arm propensity overlap, outcome-model calibration, and effective sample size.

eFigure S6. Exploratory depth-2 policy tree summarizing heterogeneous treatment effects in MIMIC-IV.

eFigure S7. Heatmap of S3 versus S1 risk differences across the primary and six prespecified sensitivity specifications in MIMIC-IV (A) and eICU-CRD (B).

eFigure S8. Bootstrap risk-difference distributions for the four cohort-level doubly-robust contrasts in MIMIC-IV and eICU-CRD

**eMethods S1. Sepsis-3 septic shock definition and the five-component modified SOFA (mSOFA-5) score**

We applied the Sepsis-3 consensus criteria of Singer and colleagues [12], defining sepsis as suspected or documented infection with an acute change in Sequential Organ Failure Assessment (SOFA) score of 2 or more points, and septic shock as the subset requiring vasopressors to maintain mean arterial pressure (MAP) at or above 65 mmHg in the presence of serum lactate above 2 mmol/L despite adequate fluid resuscitation.

The cardiovascular component of the SOFA score was omitted from mSOFA-5 because it is derived from mean arterial pressure and vasopressor dose. Both quantities define the exposure and the shock entry criteria of this emulation, and retaining the component would condition the analysis on the treatment. The remaining five components (respiratory, coagulation, hepatic, renal, and central nervous system) were summed with the standard per-component SOFA cut-points to form mSOFA-5. The respiratory component was scored from PaO₂/FiO₂ with the conventional thresholds; arterial blood gases were recorded in approximately 62.5% of stays, and a missing value was scored as normal (0). Respiratory severity in patients without arterial sampling was instead represented by the SpO₂/FiO₂ ratio, entered as a separate continuous baseline covariate.

mSOFA-5 was used at two time points for two purposes. As a Sepsis-3 entry trigger it was evaluated at organ-dysfunction onset (the mSOFA-5 onset trigger), where an acute increase of two or more points within 48 hours of admission, relative to an assumed baseline of zero, met the organ-dysfunction criterion; in this septic-shock cohort that increase is reached at or near ICU admission, so cohort entry is governed by the shock triad rather than by the two-point threshold. As a baseline severity covariate it was evaluated at T₀ as the worst value within the preceding six hours. Because cumulative urine output is unreliable when vasopressors are initiated, little having accrued since admission and patients being misclassified as oliguric, the renal component of the T₀ covariate was scored from serum creatinine.

Suspected infection was defined by concurrent administration of intravenous antibiotics and ordering of bacterial or fungal cultures within a 24-hour window, following the operationalization of Seymour and colleagues [25]. The acute fluid resuscitation criterion required either at least 30 mL/kg of crystalloid in the 6 hours preceding time zero or initiation of any acute vasopressor for at least one continuous hour. Time zero (T₀) was the first hour at which the shock triad was met in a patient with suspected infection and established organ dysfunction. The shock triad comprised mean arterial pressure below 65 mmHg, lactate above 2 mmol/L, and either at least 30 mL/kg of crystalloid in the preceding six hours or initiation of a vasopressor for at least one continuous hour. Because the two-point organ-dysfunction increase is satisfied at or near ICU admission in this cohort, the shock triad is the operative entry criterion.

**eMethods S2. Norepinephrine-equivalent (NEE) dose conversion**

Total vasopressor exposure was expressed in norepinephrine-equivalent dose (NEE), in units of µg/kg/min. The conversion factors followed the Society of Critical Care Medicine and European Society of Intensive Care Medicine joint task force position paper of Wieruszewski and colleagues [13]. Norepinephrine and epinephrine were treated as equipotent on a 1:1 mass basis. Vasopressin, infused at a fixed rate of 0.03 U/min in the absence of weight adjustment, was converted to a norepinephrine equivalent of approximately 0.1 µg/kg/min at a 70 kg reference weight, then scaled by actual body weight where available. Phenylephrine, administered as a first-line agent in a small fraction of post-anesthesia patients and used only in a sensitivity analysis, was converted at a 1:10 ratio (10 µg of phenylephrine equivalent to 1 µg of norepinephrine).

NEE at T₀ was computed as the instantaneous sum across all active continuous infusions, using the most recent infusion rate within the hour preceding T₀. Pre-T₀ peak NEE was computed as the maximum hourly NEE across the 6 hours preceding T₀. Both quantities entered the propensity and outcome models as continuous covariates.

**eMethods S3. Baseline covariate definitions, missingness assessment and missing-at-random justification**

Twenty-four baseline covariates were extracted at T₀ or summarized over the 6 hours preceding T₀, and entered the propensity and outcome models as a fixed prespecified set:

(i) Demographics: age (years), sex (male/female), self-reported race or ethnicity (white, Black, Hispanic, Asian, other or unknown), admission weight (kg), and body mass index (BMI, kg/m²).

(ii) Pre-existing comorbidities, identified by ICD-9-CM and ICD-10-CM codes recorded at any prior admission or as a present-on-admission diagnosis: congestive heart failure, chronic kidney disease of stage 3 or higher (estimated glomerular filtration rate [eGFR] <60 mL/min/1.73 m² for ≥3 months, or coded diagnosis), chronic liver disease (cirrhosis, viral hepatitis, alcoholic liver disease), and diabetes mellitus (any type).

(iii) Acute severity at T₀: mSOFA-5 (evaluated at T₀ as defined in eMethods S1), MAP (mmHg), arterial lactate (mmol/L), NEE (µg/kg/min), mechanical ventilation status (present or absent), and the SpO₂/FiO₂ ratio. Haemodynamic and laboratory values were taken as the measurement most proximate to T₀ within the preceding hour.

(iv) Pre-T₀ trajectory features: 6-hour pre-T₀ minimum MAP, mean MAP, mean lactate, peak lactate, mean NEE, and peak NEE.

(v) Time intervals: hours from suspected-infection identification to first organ dysfunction onset, and from first organ dysfunction onset to T₀.

(vi) Empirical antibiotic class active at T₀, coded as a five-level categorical variable (vancomycin; piperacillin-tazobactam; levofloxacin; ceftriaxone; other or unknown), based on the agent administered closest to T₀.

(vii) Outcome ascertainment and censoring. The primary outcome was in-hospital mortality, with administrative follow-up censored at 14 days from T₀. Discharge alive to home, rehabilitation, or skilled-nursing facility, and continued admission at end of follow-up, were coded as alive. Patients transferred to another acute hospital and patients leaving against medical advice were treated as right-censored, with inverse-probability-of-censoring weights estimated from a Cox proportional-hazards model fit on the 24 baseline covariates listed above. Weights were truncated at the 99th percentile to limit the influence of extreme values. Discharges to hospice were coded as alive in the primary analysis; a prespecified sensitivity analysis recoded these as deaths.

Operational source mappings to MIMIC-IV (chartevents, labevents, prescriptions, inputevents, diagnoses_icd, admissions) and to eICU-CRD (vitalperiodic, lab, infusiondrug, medication, diagnosis, patient) are listed in the attached covariate dictionary, available with the analysis code.

Pairwise correlations among continuous covariates are shown in eFigure S2; off-diagonal correlations exceeding |0.85| occurred only between mathematically or measurement-derived pairs (weight and body mass index; mean and maximum within the same 6-hour trajectory window for lactate, NEE and MAP), which were retained on physiological grounds; all remaining pairs had |r| < 0.50, supporting the use of all covariates jointly in the nuisance models without further screening. The pattern of missingness across covariates is shown in eFigure S3.

Missingness exceeding 20% was confined to BMI (driven by sporadic recording of admission height), the lactate trajectory features (driven by clinician-discretion sampling of arterial blood gases), and—in eICU-CRD only—the MAP trajectory features (driven by gaps in vital-sign recording across contributor hospitals). For each of these variables, missingness was patterned on the frequency of clinical measurement rather than on the underlying value of the missing variable or on the outcome. Specifically, BMI missingness was independent of measured covariates after conditioning on hospital and ICU type. Lactate-trajectory missingness reflected variation in arterial-line placement and in clinician thresholds for arterial blood-gas sampling, both of which were captured by the pre-T₀ severity covariates included in the imputation model. MAP-trajectory missingness in eICU-CRD reflected database-level vital-sign archival gaps. We therefore proceeded under a missing-at-random (MAR) assumption with respect to the joint set of measured covariates, and quantified sensitivity to this assumption through the prespecified Rubin-pooled MICE imputation analysis (eMethods S7). Imputation procedure. For the primary analysis, continuous variables were imputed by a single deterministic round-robin iterative regression (scikit-learn IterativeImputer; 20 iterations, random seed 42), binary variables by the most frequent category, and categorical variables by an explicit "Other" bucket. As a prespecified sensitivity analysis, multiple imputation by chained equations (MICE; m = 5 imputations, Bayesian-ridge posterior draws, Rubin's-rules pooling) was performed, with the fraction of missing information reported per anchor.

**eMethods S4. Cross-fitted doubly-robust learner : outcome and propensity model formulations**

Let A ∈ {S1, S2, S3} denote the assigned strategy, X the vector of baseline covariates listed in eMethods S3, and Y the in-hospital mortality indicator. The conditional outcome regression was specified as

µₐ(X) = E[Y | A = a, X], for each a ∈ {S1, S2, S3},

and the propensity score as the multinomial logistic model

eₐ(X) = P(A = a | X).

The doubly-robust pseudo-outcome for contrast a versus reference a' was defined as

φₐ,ₐ′(X, A, Y) = µₐ(X) − µₐ′(X) + 1{A = a}(Y − µₐ(X))/eₐ(X) − 1{A = a′}(Y − µₐ′(X))/eₐ′(X),

following the augmented inverse-probability-weighted form. The average treatment effect for contrast a versus a′ was estimated as the empirical mean of φₐ,ₐ′ across the trimmed cohort.

Estimation used a 5-fold cross-fitting scheme. The cohort was randomly partitioned into five folds. For each held-out fold k, both nuisance components—the propensity score eₐ(X) and the per-strategy outcome regression µₐ(X)—were fit on the remaining four folds, then evaluated on fold k to construct the φ values for fold-k patients. The procedure was repeated for k = 1, …, 5, so that every patient received a φ value computed from nuisance models that did not see that patient during fitting.

The propensity model was a multinomial logistic regression with all 24 covariates entered linearly, fit using L-BFGS optimization with default scikit-learn convergence tolerance. The per-strategy outcome model was an L2-regularized logistic regression with the regularization strength selected by an inner 3-fold cross-validation grid search over C ∈ {0.01, 0.1, 1, 10}, scoring by negative log-likelihood. We chose L2-regularized logistic regression as the primary outcome model after pilot calibration assessment showed superior small-subset calibration relative to gradient-boosted trees, particularly within the smaller S2 and S3 strata (eFigure S4); a gradient-boosted outcome model was retained as a prespecified sensitivity specification.

**eMethods S5. Bootstrap protocol and cross-implementation numerical check**

Confidence intervals and two-sided p-values for each contrast were obtained by 2,000 patient-level bootstrap resamples drawn with replacement from the trimmed cohort, with random seed set to 42. A single harmonized procedure was applied to both databases: in MIMIC-IV (n = 2,415) and in eICU-CRD (n = 568), the full analytic pipeline—nuisance fitting under 5-fold cross-fitting, propensity trimming, and computation of the doubly-robust contrast—was rerun from scratch on the resampled rows of each replicate. The 95% confidence interval was computed from the 2,000 resampled contrast estimates by the bias-corrected and accelerated (BCa) method; the two-sided p-value was twice the smaller of the proportion of replicates above and below zero, capped at 1.0.

Bootstrap resampling was conducted at the patient level rather than the fold level, so that fold assignment was re-randomized within each replicate; this captures variability arising from both finite-sample sampling and from the cross-fitting partition. The bootstrap point estimate (mean of the 2000 replicates) is reported in Table 2 alongside the original-sample point estimate; differences below 1 percentage point across all four cohort-level contrasts indicated absence of bootstrap-induced bias.

To verify numerical correctness of the in-house implementation, the cohort-level S3 versus S1 contrast in MIMIC-IV was independently re-estimated using a separate doubly-robust implementation with identical nuisance-model specifications (multinomial-logistic propensity and per-strategy L2-regularized logistic outcome under 5-fold cross-fitting) and the same hard-trimming rule, with the doubly-robust scores averaged over the identical set of trimmed patients. The two implementations agreed to within 0.3 percentage points on the point estimate and 0.5 percentage points on the lower confidence bound, confirming that the bootstrap protocol and pseudo-outcome computation were not affected by implementation-specific bugs.

**eMethods S6. Policy tree fitting, honest cross-fit policy values and bootstrap stability**

A depth-2 policy tree was fit to the matrix of doubly-robust (DR) scores using the policytree R package (version 1.2.4) of Sverdrup, Kanodia, Zhou, Athey and Wager [10]. Input to the policytree::policy_tree function was the n × 3 matrix of DR scores (one column per strategy), with the corresponding patient covariate matrix as candidate splitting variables. The minimum node size was set to 5% of the trimmed sample, and the candidate splitting set comprised all 24 baseline covariates listed in eMethods S3. The tree maximizes the empirical sum of DR scores across patients assigned to each leaf, and its leaves return one of the three candidate strategies.

Honest cross-fit policy values were computed by 5-fold cross-fitting at the policy-evaluation level. The cohort was partitioned into five evaluation folds. For each held-out fold k, the policy tree was re-fit on the remaining four folds, then applied to assign a strategy to each patient in fold k; the counterfactual mortality of the resulting policy was estimated by the average of fold-k DR scores selected by the policy assignment. Cross-fit values were averaged across folds to yield a single counterfactual-mortality estimate, with 95% bias-corrected and accelerated (BCa) confidence interval from the 2,000 patient-level bootstrap resamples described in eMethods S5. The same procedure was used to evaluate each "always-Sₐ" reference rule (a ∈ {S1, S2, S3}) and the observed clinician policy. Two-sided p-values for each rule against the clinician policy were computed as twice the smaller of the proportion of bootstrap replicates in which the rule's counterfactual mortality exceeded that of the clinician policy and the proportion in which it was lower, capped at 1.0, using the same bootstrap convention as eMethods S5.

Bootstrap stability of the tree's structure was assessed by re-fitting the depth-2 policy tree on each of 2,000 patient-level bootstrap resamples and recording the modal top-split variable and the per-patient assignment. The modal top-split variable across resamples is reported in the Discussion; per-patient plurality agreement was computed as the median, across patients, of the proportion of bootstrap trees that assigned the same strategy as the primary fit. The in-sample (non-honest) policy-tree value—the average DR score selected by the tree fit and evaluated on the same data—was also reported, and the gap between the in-sample and the cross-fit estimate quantifies the optimism induced by overfitting (eTable S4).

**eMethods S7. Prespecified sensitivity specifications and E-value formula**

Six sensitivity specifications were prespecified before unblinding any external-replication estimate:

(i) Stricter propensity trimming, with retained propensity range narrowed to π ∈ [0.05, 0.95];

(ii) No propensity trimming, with all eligible patients retained regardless of estimated propensity;

(iii) Gradient-boosted outcome model (scikit-learn HistGradientBoostingClassifier with default hyperparameters), substituted for L2-regularized logistic regression in the outcome nuisance;

(iv) Widened strategy-assignment window, with strategy assigned by exposures observed within [T₀, T₀+6 h] rather than [T₀, T₀+3 h];

(v) Addition of a fourth phenylephrine-only arm, in which patients who received phenylephrine as a first-line agent without subsequent norepinephrine within the assignment window were retained as a separate strategy and included in the multinomial propensity model;

(vi) Rubin-pooled multiple imputation by chained equations (MICE), with m = 5 imputations generated under a Bayesian-ridge per-feature posterior, the full doubly-robust pipeline rerun on each imputed dataset, and point estimate, variance and degrees of freedom pooled across imputations using Rubin's rules.

For sensitivity to unmeasured confounding, E-values were computed for the primary anchor contrast (S3 versus S1 at NEE <0.25 µg/kg/min) and for the age ≥65 secondary anchor, following VanderWeele and Ding [16]. The risk-ratio scale point estimate RR was approximated from the risk-difference estimate via RR = 1 + Δ̂/p₀, where Δ̂ is the doubly-robust risk-difference point estimate and p₀ is the observed event rate in the reference (S1) stratum. The E-value for the point estimate was then

E_value(RR) = RR + sqrt( RR · (RR − 1) ),

for RR > 1, and E_value(1/RR) for RR < 1. The E-value for the confidence-interval bound nearest the null was computed by substituting the corresponding bound of the 95% CI for RR. The interpretation is the minimum strength of association, on the risk-ratio scale, that an unmeasured confounder would need to have with both treatment assignment and outcome to fully explain away the observed effect, conditional on measured covariates.

**eTable S1. Cohort-level outcomes by treatment strategy in MIMIC-IV and eICU-CRD.**

| **Database** | **Group** | **n** | **In-hospital mortality, %** | **30-day mortality, %** | **28-day RRT, %** | **ICU LOS, h, median** | **Ventilator-free days, median** | **Shock duration, h, median** | **Shock resolved, %** |
| --- | --- | --- | --- | --- | --- | --- | --- | --- | --- |
| MIMIC-IV | S1 (NE alone) | 1915 | 38.12 | 41.46 | 20.26 | 124.1 | 25 | 32.0 | 82.82 |
| MIMIC-IV | S2 (NE + vasopressin) | 751 | 53.79 | 55.66 | 36.09 | 159.9 | 0 | 60.0 | 68.71 |
| MIMIC-IV | S3 (NE + epinephrine) | 369 | 44.72 | 44.72 | 33.88 | 132.9 | 24 | 52.5 | 69.92 |
| MIMIC-IV | Censored (alternative first-line agent) | 1148 | 20.64 | 20.99 | 14.11 | 96.3 | 28 | 16.0 | 90.24 |
| eICU-CRD | S1 (NE alone) | 440 | 40.68 | 40.45 | 11.59 | 99.7 | 28 | 17.0 | 82.50 |
| eICU-CRD | S2 (NE + vasopressin) | 93 | 55.91 | 55.91 | 25.81 | 121.1 | 0 | 32.0 | 82.80 |
| eICU-CRD | S3 (NE + epinephrine) | 73 | 42.47 | 42.47 | 23.29 | 78.4 | 28 | 27.0 | 78.08 |
| eICU-CRD | Censored (alternative first-line agent) | 80 | 37.50 | 36.25 | 17.50 | 135.4 | 28 | 19.5 | 92.50 |

Cohort-level clinical outcomes for the norepinephrine-anchored cohort prior to propensity trimming, presented separately for MIMIC-IV and eICU-CRD. The S1 / S2 / S3 rows correspond to the three pre-specified treatment strategies; the censored row aggregates patients who received an alternative first-line vasopressor (most often phenylephrine in post-anesthesia contexts, or dopamine, vasopressin or epinephrine without subsequent norepinephrine within the strategy-assignment window) and were therefore excluded from the three-arm comparison. In-hospital and 30-day mortality are reported as percentages; ICU length of stay, ventilator-free days at 28 days, and shock duration are reported as medians; 28-day renal replacement therapy and shock-resolution proportion are reported as percentages. Cohort sizes for the trimmed three-arm analytic cohort (after propensity trimming) are reported in Table 1; the smaller analytic-cohort counts (MIMIC-IV: S1 1,394, S2 683, S3 338; eICU-CRD: S1 407, S2 90, S3 71 ) reflect removal of patients with estimated propensity outside [0.01, 0.99]. eICU-CRD, eICU Collaborative Research Database; ICU, intensive care unit; LOS, length of stay; MIMIC-IV, Medical Information Mart for Intensive Care IV; NE, norepinephrine; RRT, renal replacement therapy; S1, norepinephrine alone; S2, norepinephrine plus vasopressin; S3, norepinephrine plus epinephrine.

**eTable S2. Subgroup heterogeneity of average treatment effects in MIMIC-IV — long format.**

| **Subgroup** | **Level** | **n** | **Contrast** | **ATE, pp (95% CI)** | **p** |
| --- | --- | --- | --- | --- | --- |
| NEE at T₀, µg/kg/min | <0.25 | 1287 | S3 vs S1 | −10.87 (−19.66 to +2.97) | 0.097 |
|  | ≥0.25 | 1128 | S3 vs S1 | −8.28 (−24.86 to +6.11) | 0.217 |
|  | <0.25 | 1287 | S2 vs S1 | +7.26 (−0.54 to +15.55) | 0.073 |
|  | ≥0.25 | 1128 | S2 vs S1 | −4.85 (−20.06 to −0.51) | 0.039 |
| Lactate at T₀, mmol/L | <4 | 1494 | S3 vs S1 | −12.53 (−23.40 to +0.78) | 0.068 |
|  | ≥4 | 921 | S3 vs S1 | −5.01 (−23.59 to +8.51) | 0.384 |
|  | <4 | 1494 | S2 vs S1 | +7.75 (−1.16 to +14.63) | 0.081 |
|  | ≥4 | 921 | S2 vs S1 | −8.37 (−25.66 to −3.10) | 0.012 |
| Age, years | <65 | 1017 | S3 vs S1 | −0.03 (−16.37 to +14.66) | 0.982 |
|  | ≥65 | 1398 | S3 vs S1 | −16.67 (−28.65 to −4.36) | 0.011 |
|  | <65 | 1017 | S2 vs S1 | +1.92 (−15.04 to +9.70) | 0.719 |
|  | ≥65 | 1398 | S2 vs S1 | +1.38 (−8.44 to +7.18) | 0.903 |
| Chronic kidney disease, stage ≥3 | No | 1738 | S3 vs S1 | −7.64 (−17.89 to +5.74) | 0.289 |
|  | Yes | 677 | S3 vs S1 | −14.85 (−37.33 to −2.11) | 0.029 |
|  | No | 1738 | S2 vs S1 | +1.35 (−8.85 to +7.27) | 0.891 |
|  | Yes | 677 | S2 vs S1 | +2.26 (−12.86 to +9.67) | 0.738 |
| Chronic liver disease | No | 2073 | S3 vs S1 | −13.98 (−26.18 to −6.11) | 0.001 |
|  | Yes | 342 | S3 vs S1 | Not estimable | — |
|  | No | 2073 | S2 vs S1 | +1.38 (−11.11 to +5.97) | 0.727 |
|  | Yes | 342 | S2 vs S1 | +2.95 (−16.63 to +15.95) | 0.912 |

Long-format presentation of the subgroup heterogeneity estimates summarized in Table 3 of the main text. Each row reports a single (subgroup × level × contrast) combination from the trimmed MIMIC-IV cohort (n = 2,415). Effects are expressed in percentage points (pp) as risk differences for in-hospital mortality; positive values indicate higher mortality versus the reference S1, negative values indicate lower mortality versus S1. Point estimates, 95% confidence intervals and two-sided p-values were obtained from 2,000 patient-level bias-corrected and accelerated (BCa) bootstrap resamples (random seed 42) of the cross-fitted doubly-robust learner. Within each subgroup, the S3 versus S1 contrast is presented before the S2 versus S1 contrast. The S3 versus S1 contrast in the chronic-liver-disease (Yes) stratum was not estimable because the corresponding S3 cell contained only 27 patients (eTable S3). Per-strategy cell sizes within each subgroup level are reported in eTable S3. ATE, average treatment effect; CI, confidence interval; NEE, norepinephrine-equivalent dose; pp, percentage points; S1, norepinephrine alone; S2, norepinephrine plus vasopressin; S3, norepinephrine plus epinephrine; T₀, time zero of septic shock onset.

**eTable S3. Per-strategy cell sizes within each prespecified subgroup level (MIMIC-IV trimmed cohort).**

| **Subgroup** | **Level** | **n** | **S1** | **S2** | **S3** |
| --- | --- | --- | --- | --- | --- |
| NEE at T₀, µg/kg/min | <0.25 | 1287 | 990 | 179 | 118 |
|  | ≥0.25 | 1128 | 404 | 504 | 220 |
| Lactate at T₀, mmol/L | <4 | 1494 | 983 | 384 | 127 |
|  | ≥4 | 921 | 411 | 299 | 211 |
| Age, years | <65 | 1017 | 551 | 331 | 135 |
|  | ≥65 | 1398 | 843 | 352 | 203 |
| Chronic kidney disease, stage ≥3 | No | 1738 | 1000 | 507 | 231 |
|  | Yes | 677 | 394 | 176 | 107 |
| Chronic liver disease | No | 2073 | 1194 | 568 | 311 |
|  | Yes | 342 | 200 | 115 | 27 |

Sample size per treatment strategy within each level of the five prespecified subgroups, in the trimmed MIMIC-IV cohort (n = 2,415). Rows in each subgroup sum to the level total (n column), and the two levels of each subgroup sum to 2,415. The S3 cell of the chronic-liver-disease (Yes) stratum contained 27 patients, below the threshold for stable doubly-robust estimation, and the corresponding S3 versus S1 contrast was reported as not estimable in Table 3 and eTable S2. NEE, norepinephrine-equivalent dose; S1, norepinephrine alone; S2, norepinephrine plus vasopressin; S3, norepinephrine plus epinephrine; T₀, time zero of septic shock onset.

**eTable S4.** **In-sample versus honest cross-fit counterfactual mortality and optimism gap for each candidate policy (MIMIC-IV).**

| **Policy** | **In-sample (non-honest)** | | | **Honest 5-fold cross-fit** | | | **Optimism gap, pp** |
| --- | --- | --- | --- | --- | --- | --- | --- |
|  | **Mortality, %** | **95% CI** | **p vs clinician** | **Mortality, %** | **95% CI** | **p vs clinician** |  |
| Clinician (observed policy) | 47.31 | (40.70 to 56.77) | — | 47.31 | (40.70 to 56.77) | — | 0.00 |
| Always S1 (NE alone) | 46.67 | (43.48 to 54.92) | 0.908 | 46.67 | (43.48 to 54.92) | 0.908 | 0.00 |
| Always S2 (NE + vasopressin) | 48.28 | (41.92 to 51.49) | 0.847 | 48.28 | (41.92 to 51.49) | 0.847 | 0.00 |
| Always S3 (NE + epinephrine) | 37.01 | (29.43 to 47.59) | 0.002 | 37.01 | (29.43 to 47.59) | 0.002 | 0.00 |
| Policy tree (depth 2) | 27.87 | (18.73 to 32.34) | <0.001 | 37.89 | not shown | — | +10.02 |

Counterfactual in-hospital mortality of each candidate policy in the trimmed MIMIC-IV cohort (n = 2,415), reported under two estimators. The in-sample (non-honest) estimator fits and evaluates the policy on the same data; the honest 5-fold cross-fit estimator fits the policy on four folds and evaluates on the held-out fifth fold, repeated across folds. The optimism gap is the cross-fit estimate minus the in-sample estimate, in percentage points; a positive value indicates that the in-sample estimator is overoptimistic relative to the honest estimator. By construction, the four fixed-strategy and clinician-observed policies do not overfit, so their in-sample and honest estimates coincide and the optimism gap is zero; the depth-2 policy tree shows an optimism gap of +10.02 pp, reflecting overfitting of the tree to the same data on which it was evaluated. The in-sample policy-tree confidence interval is shown for descriptive purposes only and does not constitute a valid inferential interval, because the policy tree is data-adaptive and is refit on every bootstrap resample; for the honest policy-tree value — the inferential policy value reported in Table 4 — the confidence interval and p-value are therefore omitted ("not shown"/"—"). The honest cross-fit column reproduces Table 4 of the main text. Point estimates and 95% confidence intervals are from 2,000 patient-level bias-corrected and accelerated (BCa) bootstrap resamples (random seed 42); two-sided p-values compare each policy against the clinician-observed policy using the same bootstrap distribution. CI, confidence interval; MIMIC-IV, Medical Information Mart for Intensive Care IV; NE, norepinephrine; pp, percentage points; S1, norepinephrine alone; S2, norepinephrine plus vasopressin; S3, norepinephrine plus epinephrine.

**eTable S5.** **Sensitivity analyses of the doubly-robust contrast estimates (MIMIC-IV).**

| **Specification** | **n** | **Overall S2 vs S1, pp** | **Overall S3 vs S1, pp** | **Lactate <4, S3 vs S1, pp** | **NEE <0.25 (primary anchor), S3 vs S1, pp** | **Age ≥65, S3 vs S1, pp** |
| --- | --- | --- | --- | --- | --- | --- |
| **Primary specification (trim π∈[0.01, 0.99]; L2 logistic; 3-h window)** | **2415** | **+1.60** | **−9.66** | **−12.53** | **−10.87** | **−16.67** |
| (i) Stricter propensity trimming, π∈[0.05, 0.95] | 1478 | +4.32 | −10.08 | −14.27 | **−12.93** | −14.15 |
| (ii) No propensity trimming | 3035 | N/E | N/E | N/E | **−18.96** | N/E |
| (iii) Gradient-boosted outcome model | 2415 | −1.56 | −5.51 | −7.89 | **−9.49** | −10.52 |
| (iv) Widened strategy window [T₀, T₀+6 h] | 3612 | +9.22 | −5.92 | −6.34 | **−5.32** | −13.21 |
| (v) Four-arm specification with phenylephrine-only as S4 | 3576 | +8.08 | −9.10 | −9.94 | **−9.97** | −15.13 |
| (vi) Rubin-pooled MICE (m = 5) | 2417 | +2.73 | −10.61 | −14.91 | **−11.45** | −19.11 |
| (vii) Landmark analysis (follow-up from T₀+3 h) | 2396 | +2.62 | −8.68 | −10.12 | **−8.80** | −13.93 |
| (viii) Hospice discharges reclassified as deaths | 2415 | +1.56 | −10.60 | −12.38 | **−14.69** | −16.27 |
|  |  |  |  |  |  |  |
| **Alternative NEE anchor thresholds (S3 vs S1, MIMIC-IV)** | | | | | | |
| **Threshold** | **n** | **S3 vs S1, pp** | **S2 vs S1, pp** |  |  |  |
| < 0.20 | 1045 | −11.61 | +7.94 |  |  |  |
| **< 0.25 (primary anchor)** | **1287** | **−10.87** | **+7.26** |  |  |  |
| < 0.30 | 1442 | −12.02 | +6.02 |  |  |  |

Doubly-robust risk-difference point estimates, in percentage points (pp), under the primary specification, the six prespecified sensitivity specifications (i–vi; eMethods S7), and two analyses added in revision (vii–viii); the lower panel reports the primary anchor under alternative NEE thresholds. Five contrasts are reported per specification: the two cohort-level overall contrasts (S2 vs S1 and S3 vs S1) and three S3 vs S1 stratum-specific contrasts at the prespecified anchors (lactate <4 mmol/L, NEE <0.25 µg/kg/min, and age ≥65 years). The NEE <0.25 µg/kg/min column is the prespecified primary anchor. Positive values indicate higher mortality versus the reference S1; negative values indicate lower mortality versus S1. The "no propensity trimming" row reports only the primary anchor effect; the other cells are marked N/E. Row (vi) reports Rubin-pooled MICE (m = 5) point estimates; for this row, n is the mean trimmed cohort size across the five imputations (range 2,395–2,429), trimming having been performed independently within each imputation, and it therefore differs slightly from the single-imputation primary cohort (n = 2,415). The corresponding cohort-level overall MICE estimates with confidence intervals are reported in Table 2 of the main text. Row (vii) counts follow-up from T₀+3 h (landmark) to address immortal-time bias; row (viii) reclassifies hospice discharges as deaths. The lower panel re-estimates the primary anchor at NEE thresholds of <0.20, <0.25 and <0.30 µg/kg/min (eICU-CRD directionally concordant but underpowered at the lower thresholds). CI, confidence interval; L2, ridge (squared) regularization; MICE, multiple imputation by chained equations; MIMIC-IV, Medical Information Mart for Intensive Care IV; N/E, not estimated; NEE, norepinephrine-equivalent dose; pp, percentage points; S1, norepinephrine alone; S2, norepinephrine plus vasopressin; S3, norepinephrine plus epinephrine; S4, phenylephrine-only; T₀, time zero of septic shock onset.

**eTable S6. E-values on the risk-ratio scale for the prespecified anchor and complementary contrasts, in MIMIC-IV and eICU-CRD.**

| **Tier** | **Subgroup** | **Contrast** | **MIMIC-IV** | | | | **eICU-CRD** | | | | **Interpretation** |
| --- | --- | --- | --- | --- | --- | --- | --- | --- | --- | --- | --- |
|  |  |  | **p₀** | **RR** | **E-value (point)** | **E-value (CI bound)** | **p₀** | **RR** | **E-value (point)** | **E-value (CI bound)** |  |
| **Tier 1** | **NEE <0.25 µg/kg/min** | **S3 vs S1** | **0.369** | **0.705** | **2.19** | **1.00** | **0.360** | **0.619** | **2.61** | **1.00** | **Moderate point E-value; CI bound at null in both cohorts** |
| Tier 2 | Age ≥65 years | S3 vs S1 | 0.440 | 0.621 | 2.60 | 1.46 | 0.417 | 0.901 | 1.46 | 1.00 | Robust in MIMIC-IV (CI E-value 1.46); null in eICU-CRD |
| Tier 2 | Lactate <4 mmol/L | S3 vs S1 | 0.350 | 0.642 | 2.49 | 1.00 | 0.311 | 0.905 | 1.44 | 1.00 | Moderate point E-value in MIMIC-IV; CI bound at null |
| Tier 3 | Overall (full cohort) | S2 vs S1 | 0.402 | 1.040 | 1.24 | 1.00 | 0.418 | 1.152 | 1.57 | 1.00 | Inconclusive (CI crosses null) |
| Tier 3 | Overall (full cohort) | S3 vs S1 | 0.402 | 0.760 | 1.96 | 1.06 | 0.418 | 1.017 | 1.15 | 1.00 | Borderline in MIMIC-IV (CI E-value 1.06); null in eICU-CRD |
| Negative | NEE ≥0.25 µg/kg/min | S3 vs S1 | 0.483 | 0.828 | 1.71 | 1.00 | 0.573 | 1.415 | 2.18 | 1.00 | Inconclusive (negative-control stratum) |
| Negative | Lactate ≥4 mmol/L | S3 vs S1 | 0.525 | 0.905 | 1.45 | 1.00 | 0.530 | 1.076 | 1.36 | 1.00 | Inconclusive (negative-control stratum) |
| Negative | Age <65 years | S3 vs S1 | 0.343 | 0.999 | 1.03 | 1.00 | 0.418 | 1.180 | 1.64 | 1.00 | Near-null point estimate (negative-control stratum) |

E-values quantify the minimum strength of association, on the risk-ratio scale, that an unmeasured confounder would need to have with both treatment assignment and outcome to fully explain away the observed effect, conditional on the measured covariates. For each contrast, the reference event rate p₀ is the observed in-hospital mortality in the corresponding S1 stratum, and the risk ratio (RR) is approximated from the doubly-robust risk-difference point estimate using RR = (p₀ + Δ̂) / p₀ as defined in eMethods S7. The E-value for the point estimate is computed from this RR; the E-value for the confidence-interval bound is computed by substituting the bound nearest the null. An E-value of 1.00 indicates that the corresponding bound includes the null, i.e. no minimum unmeasured-confounder strength is needed to explain away that bound. Tier 1 is the prespecified primary anchor of the analysis; Tier 2 are the prespecified secondary anchors; Tier 3 are the cohort-level overall contrasts; the Negative rows are the high-severity complementary strata where no replication was prespecified. The MIMIC-IV E-values for Tier 1 (point 2.19) and the Tier 2 age ≥65 years stratum (point 2.60; CI bound 1.46) are quoted in Results §Sensitivity analyses. CI, confidence interval; eICU-CRD, eICU Collaborative Research Database; MIMIC-IV, Medical Information Mart for Intensive Care IV; NEE, norepinephrine-equivalent dose; p₀, baseline event rate in the reference S1 stratum; RR, risk ratio; S1, norepinephrine alone; S2, norepinephrine plus vasopressin; S3, norepinephrine plus epinephrine.

**eFigure S1.** Distribution of norepinephrine-equivalent dose at time zero across treatment strategies in the MIMIC-IV norepinephrine-anchored cohort.

Box plot of norepinephrine-equivalent dose (NEE) at T₀, in µg/kg/min, by treatment strategy, in the MIMIC-IV norepinephrine-anchored cohort prior to propensity trimming (S1 n = 1,915; S2 n = 751; S3 n = 369). Boxes span the interquartile range (IQR, 25th to 75th percentile); the horizontal line within each box marks the median; whiskers extend to the most extreme observations within 1.5 × IQR of the box (Tukey convention). Median NEE at T₀ rises from S1 (0.12 µg/kg/min) to S2 (0.38 µg/kg/min) and S3 (0.43 µg/kg/min), and the upper whisker extends progressively higher across S1, S2 and S3, indicating substantially greater baseline severity in patients receiving an adjunctive agent. This baseline-severity imbalance motivates the propensity-based adjustment used in the primary analysis. Per-strategy summaries from the trimmed analytic cohort are reported in Table 1; the corresponding eICU-CRD distributions are summarized numerically in Table 1 but are not shown graphically. Cohort sizes shown here correspond to the pre-trimming (NE-anchored) cohort reported in eTable S1; trimming (estimated propensity ∈ [0.01, 0.99]) yields the analytic cohort of 2,415 used in all causal estimates. IQR, interquartile range; MIMIC-IV, Medical Information Mart for Intensive Care IV; NE, norepinephrine; NEE, norepinephrine-equivalent dose; S1, norepinephrine alone; S2, norepinephrine plus vasopressin; S3, norepinephrine plus epinephrine; T₀, time zero of septic shock onset.

**eFigure S2.** Pairwise Pearson correlation matrix of continuous baseline covariates in the trimmed MIMIC-IV cohort.

Heatmap of pairwise Pearson correlation coefficients among the 16 continuous baseline covariates entered into the nuisance models, computed in the trimmed MIMIC-IV cohort (n = 2,415). Each cell reports the correlation coefficient r; cells are colored on a diverging blue–red scale from −1.00 (red) to +1.00 (blue), with white indicating r ≈ 0. Diagonal cells are 1.00 by construction. Off-diagonal correlations exceeding |0.85| occur only between mathematically or measurement-related covariates: weight and body mass index (r = 0.92, by construction); pre-T₀ mean and maximum lactate over the 6-hour window (r = 0.97); and pre-T₀ lactate trajectory features and lactate at shock onset (r = 0.92). The only other correlations above |0.50| are likewise between repeated measures of the same quantity — the within-trajectory mean–maximum or mean–minimum pairs for norepinephrine-equivalent dose and mean arterial pressure (both r = 0.79) and norepinephrine-equivalent dose at shock onset versus its pre-T₀ trajectory (r = 0.51 and 0.58); all remaining off-diagonal correlations were below |0.50|. Categorical and binary covariates (sex, race or ethnicity, comorbidities, empirical antibiotic class) are not displayed in this matrix and were retained in the nuisance models without further screening; their pairwise associations were inspected separately and showed no excessive collinearity. BMI, body mass index; MAP, mean arterial pressure; MIMIC-IV, Medical Information Mart for Intensive Care IV; mSOFA-5, modified five-component Sequential Organ Failure Assessment score; NEE, norepinephrine-equivalent dose; SpO₂/FiO₂, oxygen saturation to fraction of inspired oxygen ratio**;** T₀, time zero of septic shock onset.

**eFigure S3.** Per-covariate missingness in the MIMIC-IV norepinephrine-anchored cohort, ordered by missing rate.

Horizontal bar chart of the percent missingness for each of the 24 baseline covariates entered into the propensity and outcome models, in the MIMIC-IV norepinephrine-anchored cohort. Bars are ordered from highest to lowest missing rate; the percentage is annotated to the right of each bar. The dashed vertical line marks the 30% exclusion threshold; covariates with missingness above this threshold would have been dropped from the nuisance models. Three covariates exceeded 20% missingness in MIMIC-IV: body mass index (22.9%, driven by sporadic recording of admission height), maximum lactate over the 6-hour pre-T₀ window (21.7%) and mean lactate over the same window (21.7%, both driven by clinician-discretion sampling of arterial blood gases). Two further covariates had non-zero but moderate missingness (8.2%): minimum and mean mean-arterial pressure over the 6-hour pre-T₀ window. One additional covariate, the SpO₂/FiO₂ ratio, had 1.5% missingness. The remaining 18 covariates—including mechanical-ventilation status, which had no missing values—had no recorded missingness in MIMIC-IV. No covariate exceeded the 30% exclusion threshold; all displayed covariates were retained and missing values were imputed as described in eMethods S3. MAP, mean arterial pressure; MIMIC-IV, Medical Information Mart for Intensive Care IV; mSOFA-5, modified five-component Sequential Organ Failure Assessment score; NEE, norepinephrine-equivalent dose; T₀, time zero of septic shock onset.

**eFigure S4.** Nuisance-model diagnostics: propensity overlap and outcome-model calibration in MIMIC-IV.

Diagnostic plots for the two nuisance components of the cross-fitted doubly-robust learner, evaluated in the MIMIC-IV norepinephrine-anchored cohort (S1 n = 1,915; S2 n = 751; S3 n = 369). Panels A–C show the distribution of the multinomial-logistic propensity score for norepinephrine alone (A), norepinephrine plus vasopressin (B), and norepinephrine plus epinephrine (C), with densities for the actually received S1, S2 and S3 overlaid in blue, yellow and red, respectively; the dashed vertical lines mark the prespecified trimming bounds [0.01, 0.99]. Separation is clearest for the propensity for norepinephrine alone (A), where patients who actually received S1 cluster at high values and those who received S2 or S3 cluster lower. Panels D–F show decile calibration of the L2-regularized logistic outcome model fit per strategy (observed versus predicted in-hospital mortality) for norepinephrine alone (D; slope 0.94, intercept −0.02), norepinephrine plus vasopressin (E; slope 1.14, intercept −0.01), and norepinephrine plus epinephrine (F; slope 0.93, intercept −0.01), with the dashed diagonal indicating perfect calibration; calibration is close to ideal in all three strata, supporting the choice of L2-regularized logistic regression over gradient-boosted models for the primary specification (eMethods S4). Panels G–I summarize the inverse-probability weights by strategy: the effective sample size, ESS = (Σw)²/Σw², with the percentage of the trimmed arm in parentheses (G); the distribution of weights, defined as the inverse propensity for the assigned strategy (H); and arm size versus the number of patients trimmed (I). After trimming at [0.01, 0.99], 620 of the 3,035 norepinephrine-anchored patients were removed (S1 521, S2 68, S3 31; 2,415 retained), and the effective sample size in the trimmed cohort was S1 352 (25%), S2 418 (61%), and S3 112 (33%). Effective sample size and weights (panels G and H) are reported for the trimmed analytic cohort, because a few near-zero propensities in the full cohort would otherwise dominate the full-cohort weights; propensity overlap (A–C) and trim counts (I) are shown for the full norepinephrine-anchored cohort. ESS, effective sample size; IPW, inverse-probability weight; MIMIC-IV, Medical Information Mart for Intensive Care IV; NE, norepinephrine; S1, norepinephrine alone; S2, norepinephrine plus vasopressin; S3, norepinephrine plus epinephrine.

**eFigure S5.** Nuisance-model diagnostics in eICU-CRD: per-arm propensity overlap, outcome-model calibration, and effective sample size.

Diagnostic plots for the two nuisance components of the cross-fitted doubly-robust learner, evaluated in the eICU-CRD norepinephrine-anchored cohort (S1 n = 440; S2 n = 93; S3 n = 73). Panels A–C show the distribution of the multinomial-logistic propensity score for norepinephrine alone (A), norepinephrine plus vasopressin (B), and norepinephrine plus epinephrine (C), with densities for the actually received S1, S2 and S3 overlaid in blue, yellow and red, respectively; the dashed vertical lines mark the prespecified trimming bounds [0.01, 0.99]. Panels D–F show decile calibration of the per-strategy outcome model (observed versus predicted in-hospital mortality) for norepinephrine alone (D; slope 1.48, intercept 0.18), norepinephrine plus vasopressin (E; slope 1.39, intercept −0.11), and norepinephrine plus epinephrine (F; slope 0.89, intercept −0.02), with the diagonal indicating perfect calibration. Panels G–I summarize the inverse-probability weights by strategy: the effective sample size, ESS = (Σw)²/Σw², with the percentage of arm n in parentheses (G); the distribution of weights, defined as the inverse propensity for the assigned strategy (H); and arm size versus the number of patients trimmed (I). After trimming at [0.01, 0.99], 38 of 606 patients were removed (S1 33, S2 3, S3 2; 568 retained). Effective sample size was substantially lower in the adjunct arms (S1 284, 64% of n; S2 30, 33%; S3 13, 18%), reflecting concentrated weights (maximum weight 116.7 in S2 and 194.6 in S3). Effective sample size and weights (panels G and H) are reported for the full norepinephrine-anchored cohort before trimming, as the eICU-CRD propensities contained no near-deterministic values requiring the trimmed-cohort summary used for MIMIC-IV (eFigure S4); propensity overlap (A–C) and trim counts (I) are likewise shown for the full cohort. eICU-CRD, eICU Collaborative Research Database; ESS, effective sample size; IPW, inverse-probability weight; MIMIC-IV, Medical Information Mart for Intensive Care IV; S1, norepinephrine alone; S2, norepinephrine plus vasopressin; S3, norepinephrine plus epinephrine.

**eFigure S6. Exploratory depth-2 policy tree summarizing heterogeneous treatment effects in MIMIC-IV.**

Depth-2 policy tree fitted to the cross-fitted doubly-robust pseudo-outcome scores in the trimmed MIMIC-IV cohort (n = 2,415) using the policytree R package, with the minimum node size set to 5% of the trimmed sample (121 patients). Internal nodes show the split variable and threshold; each leaf shows the strategy assigned by the tree together with the number and percentage of patients reaching it. Strategies are S1, norepinephrine alone; S2, norepinephrine plus vasopressin; and S3, norepinephrine plus epinephrine. The tree is a descriptive, exploratory summary of estimated treatment-effect heterogeneity and is not a clinical decision rule: it is structurally unstable — across bootstrap resamples the modal first-split variable is selected in only about 20% of replicates — and several of its splits are clinically counterintuitive (e.g., older patients with low pre-T₀ peak vasopressor requirement are directed to epinephrine-containing escalation; see Discussion). NEE, norepinephrine-equivalent dose; MAP, mean arterial pressure; T₀, time zero.

**eFigure S7. Heatmap of S3 versus S1 risk differences across the primary and six prespecified sensitivity specifications in MIMIC-IV (A) and eICU-CRD (B).**

Heatmap visualization of the doubly-robust S3 versus S1 risk-difference point estimates, in percentage points (pp), at the prespecified primary anchor (norepinephrine-equivalent dose <0.25 µg/kg/min) and the two prespecified secondary anchors (lactate <4 mmol/L and age ≥65 years), under the primary specification and each of the six prespecified sensitivity specifications, shown for the derivation cohort MIMIC-IV in Panel A and the external cohort eICU-CRD in Panel B. Rows correspond to specifications: the primary specification (highlighted with a heavier border), followed by the six sensitivity specifications enumerated in eMethods S7 (stricter propensity trimming with π ∈ [0.05, 0.95]; no propensity trimming; gradient-boosted outcome model; widened strategy-assignment window of [T₀, T₀+6 h]; four-arm specification with phenylephrine-only as S4; and Rubin-pooled multiple imputation by chained equations with m = 5). Columns correspond to the three anchored contrasts. Cells are colored on a diverging blue–red scale ranging from −25 pp (deep blue) through 0 (white) to +25 pp (deep red); blue indicates lower mortality under S3 than S1, red indicates higher mortality. The numeric value in each cell is the corresponding S3 versus S1 point estimate in pp. In Panel A (MIMIC-IV), cells marked "N/E" (grey hatching) are not estimable: under the untrimmed specification, extreme propensity weights destabilise the doubly-robust estimator at the lactate and age anchors, which are therefore not reported (the NEE anchor remains estimable at −18.96 pp). All 19 estimated MIMIC-IV cells are negative, with magnitudes from −5.32 pp (NEE <0.25 anchor under the 6-hour-window specification) to −19.11 pp (age ≥65 anchor under the multiple-imputation specification), indicating that the lower mortality under S3 in MIMIC-IV is directionally robust across all specifications. In Panel B (eICU-CRD), all cells are estimable but considerably more variable, reflecting the smaller external subgroups: the NEE <0.25 anchor is negative in six of seven specifications and the age ≥65 anchor in all seven, whereas the lactate <4 anchor is unstable and changes sign across specifications; one eICU-CRD cell (−32.37 pp under the gradient-boosted outcome model) falls below the −25 pp scale limit and is shown at the limit with its value annotated. The corresponding MIMIC-IV numerical values are reported in eTable S5, and the eICU-CRD values are shown in Panel B. eICU-CRD, eICU Collaborative Research Database; MICE, multiple imputation by chained equations; MIMIC-IV, Medical Information Mart for Intensive Care IV; N/E, not estimable; NEE, norepinephrine-equivalent dose; pp, percentage points; S1, norepinephrine alone; S3, norepinephrine plus epinephrine; S4, phenylephrine-only; T₀, time zero of septic shock onset.

**eFigure S8.** Bootstrap risk-difference distributions for the four cohort-level doubly-robust contrasts in MIMIC-IV and eICU-CRD.

Each panel displays the empirical distribution of 2,000 patient-level bootstrap estimates of the cross-fitted doubly-robust risk difference (in percentage points, pp) for one of the four cohort-level contrasts: norepinephrine plus vasopressin versus norepinephrine alone (S2 vs S1) and norepinephrine plus epinephrine versus norepinephrine alone (S3 vs S1), in MIMIC-IV (top row, n = 2,415) and eICU-CRD (bottom row, n = 568). A single harmonized procedure was used for both cohorts, with the full analytic pipeline — including the propensity and outcome nuisance models — refit on each resample. The solid blue vertical line marks the original-sample point estimate; the dashed grey vertical line marks the null effect (RD = 0); the shaded blue band marks the 95% bias-corrected and accelerated (BCa) confidence interval computed from the same 2,000-resample bootstrap, so that the interval and the displayed histogram derive from a single procedure. Point estimates, BCa 95% CIs and two-sided p-values are annotated in each panel. S1, norepinephrine alone; S2, norepinephrine plus vasopressin; S3, norepinephrine plus epinephrine; BCa, bias-corrected and accelerated; pp, percentage points; RD, risk difference.
